# Chromosome-scale assembly of the *Kandelia obovata* genome

**DOI:** 10.1038/s41438-020-0300-x

**Published:** 2020-05-02

**Authors:** Min-Jie Hu, Wei-Hong Sun, Wen-Chieh Tsai, Shuang Xiang, Xing-Kai Lai, De-Qiang Chen, Xue-Die Liu, Yi-Fan Wang, Yi-Xun Le, Si-Ming Chen, Di-Yang Zhang, Xia Yu, Wen-Qi Hu, Zhuang Zhou, Yan-Qiong Chen, Shuang-Quan Zou, Zhong-Jian Liu

**Affiliations:** 1 Key Laboratory of Humid Sub-tropical Eco-Geographical Processes of the Ministry of Education, Fujian Normal University, Fuzhou, 350007 China; 2 Fujian Colleges and Universities Engineering Research Institute of Conservation and Utilization of Natural Bioresources, College of Forestry, Fujian Agriculture and Forestry University, Fuzhou, 350002 China; 3 Key Laboratory of National Forestry and Grassland Administration for Orchid Conservation and Utilization at the College of Landscape Architecture, Fujian Agriculture and Forestry University, Fuzhou, 350002 China; 4 Institute of Tropical Plant Sciences and Microbiology, National Cheng Kung University, Tainan, 701 China; 5 Administration of the Quanzhou Bay Estuary Wetland Nature Reserve, Quanzhou, 362000 China; 6 Ocean College, Minjiang University, Fuzhou, 350002 China; 7 Henry Fok College of Biology and Agriculture, Shaoguan University, Shaoguan, 512005 China

**Keywords:** Genome, Evolution

## Abstract

The mangrove *Kandelia obovata* (Rhizophoraceae) is an important coastal shelterbelt and landscape tree distributed in tropical and subtropical areas across East Asia and Southeast Asia. Herein, a chromosome-level reference genome of *K. obovata* based on PacBio, Illumina, and Hi-C data is reported. The high-quality assembled genome size is 177.99 Mb, with a contig N50 value of 5.74 Mb. A large number of contracted gene families and a small number of expanded gene families, as well as a small number of repeated sequences, may account for the small *K. obovata* genome. We found that *K. obovata* experienced two whole-genome polyploidization events: one whole-genome duplication shared with other Rhizophoreae and one shared with most eudicots (γ event). We confidently annotated 19,138 protein-coding genes in *K. obovata* and identified the MADS-box gene class and the *RPW8* gene class, which might be related to flowering and resistance to powdery mildew in *K. obovata* and *Rhizophora apiculata*, respectively. The reference *K. obovata* genome described here will be very useful for further molecular elucidation of various traits, the breeding of this coastal shelterbelt species, and evolutionary studies with related taxa.

## Introduction

Mangrove forests are coastal ecosystems with unique biodiversity that provides many ecosystem services and functions^1^. Mangrove loss will increase the threat of coastal hazards (i.e., erosion, storm surges, and tsunamis) to human safety and shoreline development^2^. Specifically, this will reduce coastal water quality and biodiversity and threaten adjacent coastal habitats, thereby weakening the main resources on which the human community relies, including a large number of products and services provided by mangroves^3,4^. Therefore, detailed studies and analyses of the genome and evolution of mangroves are urgently required, especially in the context of frequent human disturbance and inevitable sea-level rise.

**Fig. 1:**
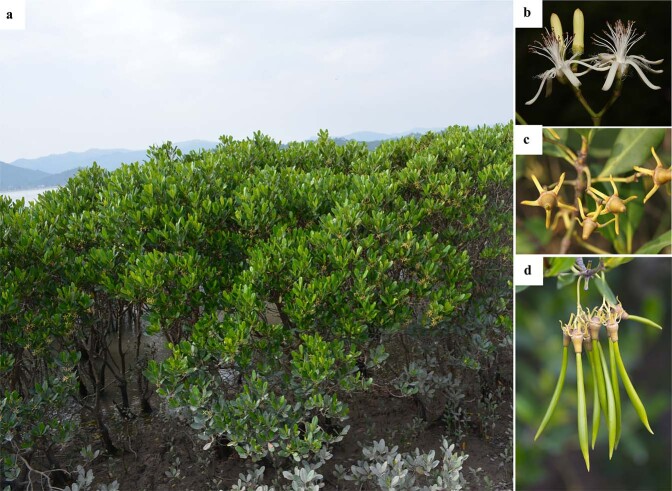
Morphological features of the flower and fruit of K. obovata. **a** *K. obovata* trees in a coastal wetland. **b** Flowers. **c** Young fruits. **d** Cone-like fruits

The mangrove species *Kandelia obovata* belongs to Rhizophoraceae, which is called “Qiuqie” in Chinese, with the Latin name of *K. candel* in “Flora Reipublicae Popularis Sinicae”^5^. Later, in 2008, its Latin name was changed to *K. obovata* in the “Flora of China”^6^. *K. obovata* is a woody plant predominantly found in tropical and subtropical tidal salt wetlands distributed from East Asia to Southeast Asia^7^. *K. obovata* adapts to transitional ecosystems where the land and ocean meet by overcoming periodic and aperiodic tidal effects, which induce high salinity, severe erosion, and anaerobic conditions^8^. *K. obovata* plays a crucial role in protecting biodiversity and combating erosion^9,10^. Specifically, the mangrove *K. obovata* can protect the embankment, accelerate the natural deposition of the beach, filter organic matter and pollutants from inland areas, and provide an ideal habitat for the marine flora and fauna^11^. At the same time, due to its beautiful shape, unique floral pattern and fragrance, *K. obovata* is an excellent coastal wetland landscape plant and horticultural ornamental plant (Fig. 1).

Here, the genome of the mangrove *K. obovata* was sequenced using PacBio sequencing as well as the Illumina next-generation sequencing platform. These data can help clarify the history of mangrove colonization and mangrove adaptation mechanisms in intertidal zones. Furthermore, this study will provide a basis for the conservation of mangrove diversity and in-depth development of genetic resources for mangroves, as well as the development and utilization of coastal horticultural plants.

## Results and discussion

### Genome sequence and assembly

**Fig. 2:**
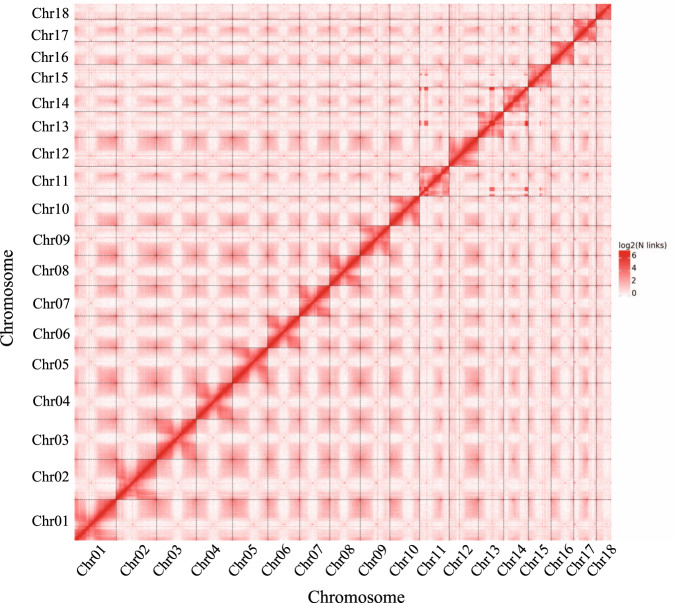
Intensity signal heat map of the Hi-C chromosome

*K. obovata* contains 36 chromosomes (2n = 2x = 36)^6^. To assess genome size, survey sequencing was performed, and 65.27 Gb of clean data was obtained (Supplementary Table 1). The survey analysis indicated that the *K. obovata* genome size is 211.86 Mb and has a low level of heterozygosity of approximately 0.38% (Supplementary Fig. 1). The assembled genome is 178.44 Mb in size, with a scaffold N50 value of 279.55 kb obtained by using Illumina sequencing (Table 1). To improve *K. obovata* assembly quality, we conducted Pacific Biosciences RSII sequencing and obtained 25 Gb of single-molecule real-time long reads (average read length of 11.9 kb; Supplementary Fig. 2, Supplementary Table 1). The final assembled genome is 177.99 Mb in size, with a contig N50 value of 5.74 Mb (Table 1). The quality of the assembly was evaluated using Benchmarking Universal Single-Copy Orthologs (BUSCO)^12^. The results showed that the gene set completeness of the assembled genome is 97.3%, indicating that the *K. obovata* genome assembly is very complete and of high quality (Table 1). Finally, high-throughput/resolution chromosome conformation capture (Hi-C) technology was adopted to assess the chromosome-level diploid genome. The results showed that the lengths of the chromosomes ranged from 5.03 to 13.8 Mb (Supplementary Table 2), with a total length of 178.01 Mb and a scaffold N50 of 10.03 Mb (Fig. 2, Table 1).

**Table 1:** The statistical results of Hi-C assembly

Assembly	Size (bp)
Illumina sequencing assembly	
Scaffold N50	279,548
Scaffold N90	28,239
Longest Scaffold	1,696,757
Total Scaffold length	178,438,058
PacBio sequencing assembly	
Contig N50	5,743,053
Contig N90	2,939,642
Longest Contig	13,452,090
Total Contig length	177,986,124
BUSCO	97.3%
Hi-C assembly	
Scaffold N50	10,026,007
Scaffold N90	7,500,541
Longest Contig	13,797,742
Total Contig length	178,014,124

### Gene prediction and annotation

We confidently annotated 19,138 protein-coding genes in *K. obovata* (Supplementary Fig. 3, Supplementary Table 3), of which 19,136 (99.17%) were supported by de novo prediction, transcriptome data, and homolog prediction (Supplementary Table 4). The genome of *Rhizophora apiculata*, also belonging to Rhizophoreae, has 26,640 protein-coding genes, which is 7502 more than observed in *K. obovata*^13^. The BUSCO^12^ assessment indicated that the completeness of the gene set of the annotated genome was 90% for *K. obovata* (Supplementary Table 5). In addition, 105 microRNAs, 307 transfer RNAs, 167 ribosomal RNAs, and 199 small nuclear RNAs were identified in the *K. obovata* genome (Supplementary Table 6).

Using homology-based and de novo approaches to identify transposable elements (TEs), we estimated that 24.07% of the *K. obovata* genome consists of repetitive sequences (Supplementary Figs. 4 and 5 and Supplementary Tables 7 and 8) and 29% of the *R. apiculata* genome consists of repetitive sequences^13^. Compared with those of closely related nonmangrove plant genomes, the repetitive portions of the *R. apiculata* genome, comprising predominantly TE families, are significantly reduced, and the decrease in TE number largely resulted in a general decrease in genome size among true mangroves^13^. The small repetitive sequences may be one reason for the small genome of *K. obovata*. In addition, 18,266 genes were functionally annotated, among which 11,124 and 14,401 were annotated to Gene Ontology terms and Kyoto Encyclopedia of Genes and Genomes terms, respectively, and 12,491 genes were functionally annotated in all five databases (Supplementary Fig. 6, Supplementary Table 9).

### Evolution of gene families

**Fig. 3:**
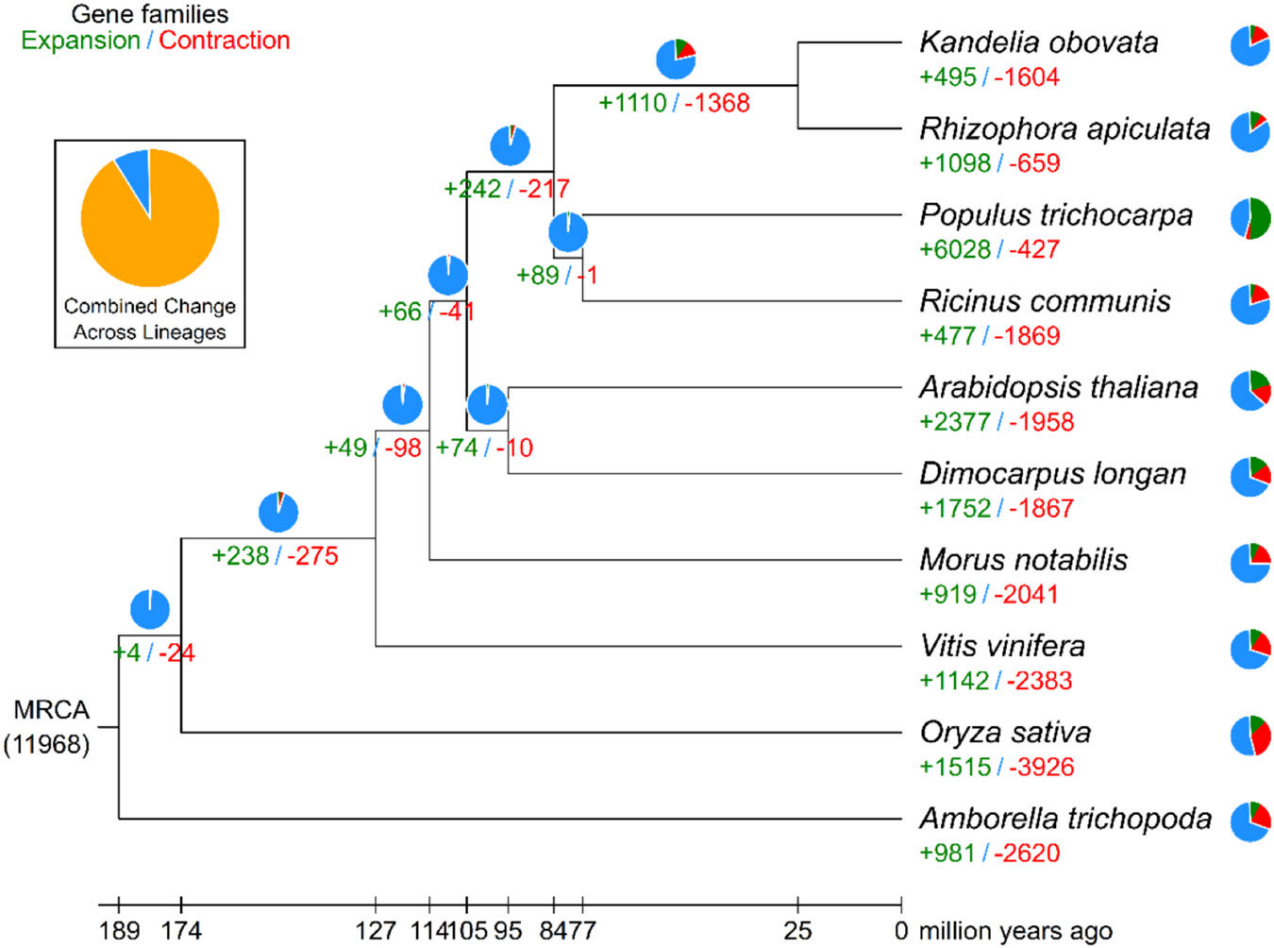
The expansion and contraction of gene families. The green number indicates the number of expanded gene families, and the red number indicates the number of contracted gene families. The blue color in the circle shows the gene families whose copy numbers are constant, while the orange color represents the proportion of 11,968 gene families in the most recent common ancestor that have expanded or contracted during late differentiation

We constructed a phylogenetic tree and estimated the divergence times of *K. obovata* and nine other plant species based on genes extracted from a total of 1095 single-copy families (Supplementary Figs. 7 and 8, Supplementary Table 10). As expected, *K. obovata* was sister to *R. apiculata* (Supplementary Fig. 9). The estimated Rhizophoreae divergence time was 83.15 Mya, and the divergence time between *K. obovata* and *R. apiculata* was 24.63 Mya (Supplementary Fig. 9). Next, using CAFÉ 3 (ref. ^14^), we found that 1110 gene families were expanded in the lineage leading to the Rhizophoreae, whereas 1368 families were contracted (Fig. 3). Four hundred and ninety-five gene families were expanded in *K. obovata*, compared with the 1098 in *R. apiculata* (Fig. 3). At the same time, 1604 gene families were contracted in *K. obovata*, compared with the 659 in *R. apiculata*. *K. obovata* has more contracted gene families than *R. apiculata* and fewer expanded gene families than *R. apiculata*, which may be the reason that the genome of *K. obovata* is smaller than that of *R. apiculata*. For the expanded gene families, we conducted GO enrichment analysis and found enrichment for the GO terms “structural constituent of cytoskeleton” and “structural constituent of ribosome” (Supplementary Table 11). For the contracted gene families, enrichment was detected for the GO terms “protein kinase activity”, “terpene synthase activity”, “oxidoreductase activity”, “nutrient reservoir activity”, “defense response”, and “sulfotransferase activity” (Supplementary Table 12). Gene families with *K. obovata*-specific expansion and contraction might relate to adaptation to *K. obovata*-specific coastal niches. Further research is required to validate the function of these genes.

### Synteny analysis and an ancient polyploidization event

Whole-genome polyploidization events are a feature of many taxa and an efficient mechanisms of genome expansion^15^. To detect the occurrence of polyploidization events in Rhizophoreae, we used the default parameters of JCVI v0.9.14 (ref. ^16^) to analyze the protein sequences of *K. obovata*, *R. apiculata*, and *Vitis vinifera* and obtained the gene pairs in the collinear regions. The results showed that there were 11,010 collinear gene pairs between *K. obovata* and *R. apiculata*, 10,893 collinear gene pairs between *K. obovata* and *V. vinifera*, 3,840 collinear gene pairs within *K. obovata* and 4,646 collinear gene pairs within *R. apiculata* (Supplementary Table 13).

**Fig. 4:**
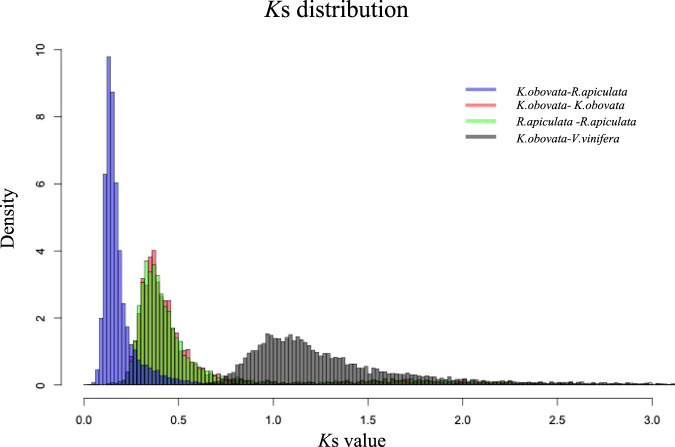
Ks distributions between *K. obovata* and *R. apiculata* and *K. obovata* and *V. vinifera* and within *K. obovata* and *R. apiculata*. Peaks of intraspecies *K*s distributions indicate ancient whole-genome polyploidization events, and peaks of interspecies *K*s distributions indicate speciation events

**Fig. 5:**
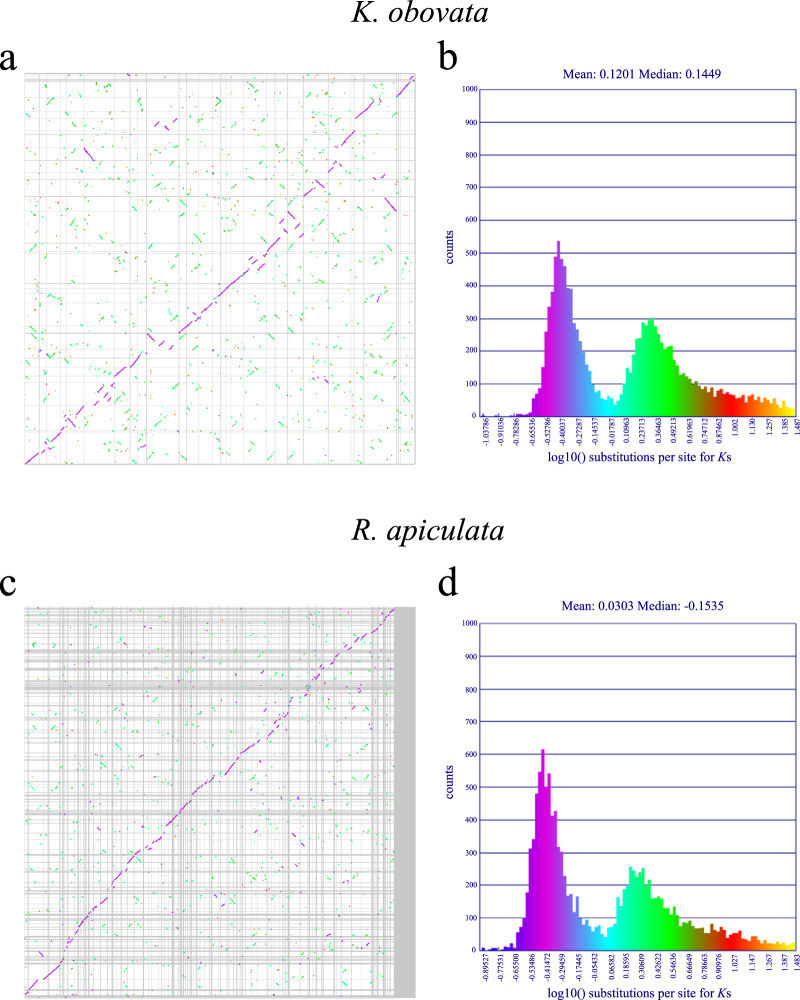
Collinear point diagram and Ks values corresponding to the collinear blocks. **a** The collinear point diagram of *K. obovata*. **b** Distribution of log10 (*K*s) values of the collinear blocks in *K. obovata*. **c** The collinear point diagram of *R. apiculata*. **d** Distribution of log10 (*K*s) values of the collinear blocks in *R. apiculata*. The ordinate of **b**, **d** is the number of gene pairs corresponding to the *K*s value, and the abscissa is the log10 (*K*s) value

We estimated the distributions of synonymous substitutions per synonymous site (*K*s) values to more precisely infer the timing of polyploidization events in the *K. obovata* genome. The distributions of *K*s for paralogous *K. obovata* genes showed two peaks, one at *K*s = 0.38 and the other at *K*s = 1.5–1.9 (Fig. 4, Supplementary Fig. 10a). The *K*s distribution of *R. apiculata* also had two peaks, one at *Ks* = 0.32 and the other at *K*s = 1.5–1.9 (Fig. 4, Supplementary Fig. 10b). The results suggested that *K. obovata* and *R. apiculata* experienced two polyploidization events. To confirm these two polyploidization events, we further analyzed the *K*s distribution of *K. obovata* and *R. apiculata* and that of *K. obovata* and *V. vinifera*. We observed that the *K*s distribution of *K. obovata* and *R. apiculata* had one peak, at *K*s = 0.1–0.16, which was smaller than the first peak in the *K*s distributions within *K. obovata* (*K*s = 0.38) and *R. apiculata* (*K*s = 0.32) (Fig. 4). The first peak in the *K. obovata K*s distribution (*K*s = 0.38) indicates that *K. obovata* shares a whole-genome duplication (WGD) event with other Rhizophoreae. In addition, we found that the *K*s distribution of *K. obovata* and *V. vinifera* had one peak, at *K*s = 0.9–1.4, which was also smaller than the second peak in the *K*s distributions within *K. obovata* (*K*s = 1.5–1.9) and *R. apiculata* (*K*s = 1.5–1.9) (Fig. 4). The second peak in the *K. obovata K*s distribution (*K*s = 1.5–1.9) indicates that the common ancestor of *K. obovata* and *V. vinifera* experienced an ancient polyploidization event. This event was shared by most eudicots, called the γ event, which is an ancient whole-genome triplication event^17^. Finally, we provide direct evidence of gene collinearity, as shown in Fig. 5; the purple peak corresponds to the first peak of the *K. obovata K*s distribution (*K*s = 0.38) and *R. apiculata K*s distribution (*K*s = 0.32) (Fig. 5b, d), and the green peak corresponds to the second peak of the *K. obovata K*s distribution (*K*s = 1.5–1.9) and *R. apiculata K*s distribution (*K*s = 1.5–1.9) (Fig. 5a, c). The purple collinear region is an extra copy of the genomes of *K. obovata* and *R. apiculata*, and the green collinear region is also an extra copy of the genes in the genomes of *K. obovata* and *R. apiculata* (Fig. 5). These copies correspond to two polyploidization events of *K. obovata* and *R. apiculata*. Therefore, our study verified that *K. obovata* experienced two polyploidization events: one WGD event shared with Rhizophoreae and one shared with most eudicots (γ event).

### MADS-box gene family analysis

**Fig. 6:**
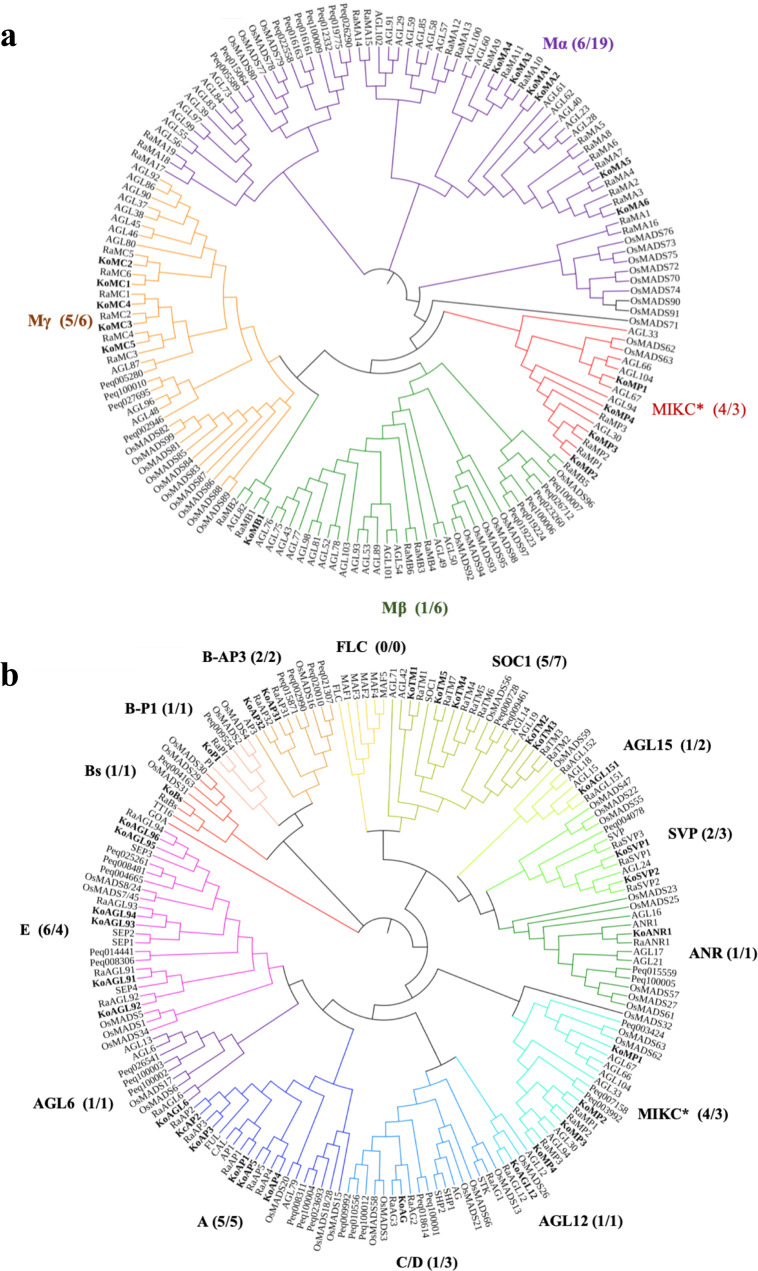
Phylogenetic analysis of MADS-box genes from *A. thaliana*, *O. sativa*, *P. equestris*, *K. obovata*, and *R. apiculata*. **a** Phylogenetic tree of type I MADS-box genes. **b** Phylogenetic tree of type II MADS-box genes. The number on the left in parentheses represents the homologous MADS genes of *K. obovata*, and the number on the right represents the homologous MADS genes of *R. apiculata*. The bolded gene ID numbers beginning with “Ko” represent the gene IDs of *K. obovata*; those beginning with “Ra” represent the gene IDs of *R. apiculata*

MADS-box genes play a key role in many important processes during plant development, especially during flower development^18^. We evaluated the MADS-box genes in *K. obovata* and *R. apiculata*. The *K. obovata* and *R. apiculata* genomes encode 43 and 65 MADS-box genes, respectively. There are 12 type I and 31 type II MADS-box genes in the *K. obovata* genome and 31 type I and 34 type II genes in the *R. apiculata* genome (Table 2, Supplementary Table 14). Interactions among type I MADS-box genes promote the initiation of endosperm development^19^. The type I genes of *R. apiculata* were approximately three times more numerous than those of *K. obovata* (Fig. 6a, Table 2). In addition, only 1 pseudogene type I genes were found in the *K. obovata* genome (Supplementary Table 14), suggesting that the type I MADS-box genes of *K. obovata* experienced a lower gain rate and higher loss rate than type II MADS-box genes.

**Table 2:** MADS-box genes in *Arabidopsis thaliana*, *Oryza sativa*, *Phalaenopsis equestris*, *K. obovata*, and *R. apiculata*

Category	*A. thaliana*^a^	*O. sativa*^b^	*P. equestris*^c^	*K. obovata*	*R. apiculata*^d^
Type II (total)	45	44	29	31	34
MIKC^c^	39	39	28	27	31
MIKC^*^	6	5	1	4	3
Type I (total)	61	31	22	12	31
Mα	25	12	10	6	19
Mβ	20	9	0	1	6
Mγ	16	10	12	5	6
Total	106	75	51	43	65

^a^The whole-genome sequence of *A. thaliana* was extracted from the NCBI database, BioProject: PRJNA477266 (ref. ^14^)

^b^The whole-genome sequence of *O. sativa* was extracted from rice.plantbiology.msu.edu/

^c^The whole-genome sequence of *P. equestris* was extracted from the NCBI database, BioProject: PRJNA192198 (ref. ^15^)

^d^The whole-genome sequence of *R. apiculata* was extracted from http://evolution.sysu.edu.cn/Sequences.html

Type II MADS-box genes include two types: MIKC^C^ and MIKC*^20^. MIKC*-type gene regulation has a major impact on pollen gene expression^21,22^. Plant MIKC^C^-type genes are the most widely studied MADS-box genes because they are essential for plant growth and development^23,24^. The *K. obovata* genome has four MIKC*-type genes and 27 MIKC^C^-type genes, while the *R. apiculata* genome has three MIKC*-type genes and 31 MIKC^C^-type genes (Fig. 6b, Table 2). Fewer *C/D*-class and *AGL6* genes were found in *K. obovata* and *R. apiculata* than in rice, whereas more *B-AP3*-class and *E*-class genes were found in *K. obovata* than in rice (Fig. 6b). *A*-class, *B*-class, *C/D*-class, and *E*-class gene clades are well known for their roles in the specification of floral organ identity^25^, notably, the ABCDE flowering model^26–28^. *K. obovata* and *R. apiculata* have the same number of *A*-class and *B*-class genes (five members). *K. obovata* (six members) has more *E*-class genes than *R. apiculata* (four members), and *R. apiculata* (one member) has fewer *C*-class genes than *K. obovata* (three members) (Fig. 6b). The *AGL12* gene is involved in root cell differentiation^29^, and the *ANR1* gene is involved in the regulation of lateral root development^30^. Furthermore, the loss of the *AGL12* gene may result in the loss of the ability to develop true roots for terrestrial growth^29^. *K. obovata* and *R. apiculata* each contain one *AGL12*-clade gene and one *ANR1*-clade gene (Fig. 6b), which may be because mangrove roots have adapted to environments at the interface of land and sea. *SOC1*, *SVP*, *FLC*, and *AGL15* regulate flowering time^31–34^. *SOC1* integrates multiple flowering signals related to photoperiod, temperature, hormones, and age^34^. Notably, we found that *SOC1*-like genes were expanded in both *K. obovata* (five members of *SOC1*) and *R. apiculata* (seven members of *SOC1*) (Fig. 6b). Sequence variation among these *SOC1*-like genes could be associated with the functional diversification of the *SOC1* clade in *K. obovata* and *R. apiculata*.

### Disease resistance-related genes

**Fig. 7:**
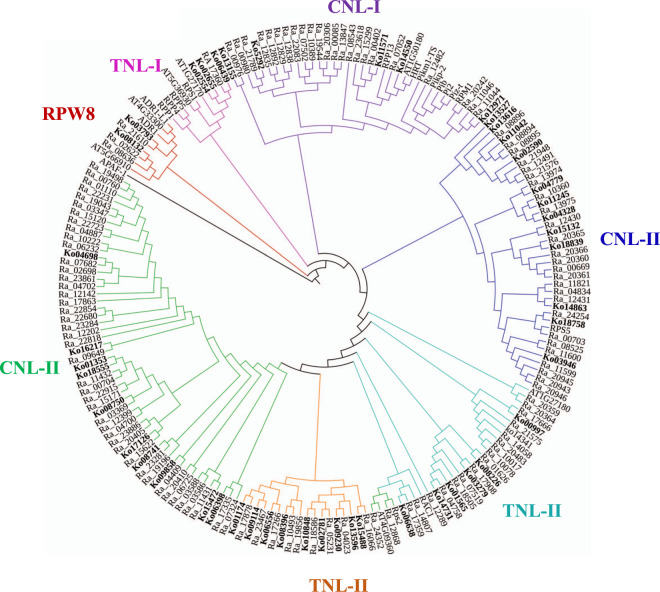
Phylogenetic reconstruction of the NLR proteins in *K. obovata* and *R. apiculata*. The NBS domain of human apoptotic protease-activating factor-1 (*APAF*-1) is located at the root of the tree. The bolded gene ID numbers beginning with “Ko” represent the gene IDs of *K. obovata*; those beginning with “Ra” represent the gene IDs of *R. apiculata*

Plant resistance genes (*R* genes) exist in large families and usually contain a nucleotide-binding site (NBS) domain and a leucine-rich repeat (LRR) domain, denoted NLR^35^. According to the presence or absence of different domains in the N-terminal region, resistance genes encoding NBS domains can be divided into the TNL (TIR-NBS-LRR), CNL (CC-NBS-LRR), and RNL (RPW8-NBS-LRR) groups^36^. A total of 165 and 292 nucleotide-binding site (NBS)-containing *R* genes were identified in *K. obovata* and *R. apiculata*, respectively; this might be because the distribution of *R. apiculata* is wider than that of *K. obovata* (Fig. 7, Supplementary Table 15).

We selected NLR candidate genes from *K. obovata* and *R. apiculata* with complete domains to construct a phylogenetic tree. The results showed that these candidate genes were divided into the TNL, RNL, and CNL families (Fig. 7). *RPW8* is a family of genes with highly specifically expressed characteristics, including resistance to powdery mildew^37^. The phylogenetic tree showed that *RPW8* genes were significantly separated from all other CNL genes (Fig. 7). The *RPW8* clade contained two *K. obovata* and three *R. apiculata* genes and clustered with two *ADR1* genes from Arabidopsis, indicating that *RPW8* genes might be associated with resistance to powdery mildew (Fig. 7).

## Conclusion

Although *K. obovata* is well known as a coastal shelterbelt and landscape tree in tropical and subtropical areas, research on this species has been hampered by a lack of genetic data. We obtained a chromosome-level reference genome of *K. obovata*, assembled a 177.99 Mb genome, and annotated 19,136 protein-coding genes. A large number of contracted gene families and a small number of expanded gene families, as well as a small number of repeated sequences, resulted in a smaller genome in *K. obovata* than in *R. apiculata*. *K*s analysis revealed that *K. obovata* experienced two polyploidization events, namely, the recent WGD shared with other Rhizophoreae and the ancient polyploidization event shared with most eudicots (γ event). The Rhizophoreae divergence time was 83.15 Mya, and the divergence time between *K. obovata* and *R. apiculata* was 24.63 Mya. We identified MADS-box and *RPW8* genes in *K. obovata*, which might be related to flowering and resistance to powdery mildew, respectively. The genomic sequence analysis of the mangrove *K. obovata* helped reveal its mechanisms of adaptation to the intertidal zone; this knowledge is critical for understanding its genetic evolution and reproduction.

## Materials and methods

### DNA preparation and sequencing

Fresh *K. obovata* tissues were collected from the Quanzhou Estuary Wetland Provincial Nature Reserve, Fujian Province, China. Genomic DNA was isolated from the fresh leaves of *K. obovata* for de novo sequencing and assembly. Paired-end libraries (500 bp) were constructed according to the Illumina protocol. Genome size and heterozygosity were measured using KmerFreq and GCE based on a 17-*K*-mer distribution. In addition, a 20 kb insert library was constructed according to the PacBio RSII protocol and subsequently sequenced on the PacBio platform (Supplementary Table 1). The transcriptomes of different tissues of *K. obovata* were sequenced on the Illumina platform.

### Genome assembly

De novo assembly of the PacBio reads was performed. FALCON (https://github.com/PacificBiosciences/FALCON)^38^ was used to correct errors in the original data. Then, SMARTdenovo v1.0 was used to assemble the corrected data^39^, and Arrow software (https://github.com/PacificBiosciences/GenomicConsensus) was used to polish the assembly results. To further eliminate Indel and SNP errors in the assembly sequence, we compared the second-generation small-fragment data to the assembly results and corrected the assembly results again with Pilon v1.22 (ref. ^40^). To confirm the quality of the genome assembly, we performed a BUSCO v3 (ref. ^12^) (http://busco.ezlab.org/) assessment using single-copy orthologous genes.

### Hi-C library construction and assembly of the chromosome

Fresh leaves of *K. obovata* were used to construct a Hi-C sequencing library, which was sequenced on the NovaSeq platform. SOAPnuke v1.5.3 (ref. ^41^) was used to filter the original data (filtration parameter: filter -n 0.01 -l 20 -q 0.4 -d -M 3 -A 0.3 -Q 2 -i -G --seqType 1) to obtain clean reads. Then, the clean data were compared with the genome using Juicer software^42^. The results were filtered, and misaligned reads were removed. The genome sequence was preliminarily clustered, sequenced, and directed using 3D-DNA^43^. Juicer-box^42^ was again used to adjust, reset, and cluster the genome sequence. Finally, we evaluated genome integrity using BUSCO v3 software^12^.

### Identification of repetitive sequences

TEs contribute to genome dynamism in terms of both size and structure through insertions and eventual loss^44^. Tandem Repeats Finder (http://tandem.bu.edu/trf/trf.html, v4.07) was used to predict tandem repeats across the genome^45^. TEs were first identified using RepeatMasker v3.3.0 (http://www.repeatmasker.org) and RepeatProteinMask based on Repbase v21.12 (http://www.girinst.org/repbase)^46^. Then, two de novo prediction software programs, RepeatModeler (http://www.repeatmasker.org/RepeatModeler/)^47^ and LTR_FINDER v1.06 (http://tlife.fudan.edu.cn/ltr_finder/)^48^, were used to identify TEs in the genomes. Finally, repeat sequences with identities ≥50% were grouped into the same classes.

### Gene prediction and annotation

Homology-based, de novo, and transcriptome-based predictions were integrated to predict high-quality protein-coding genes. For homology-based prediction, homologous proteins from five available whole-genome sequences, namely, those of *Arabidopsis thaliana*, *Linum usitatissimum*, *Populus trichocarpa*, *Ricinus communis*, and *Salix purpurea*, were aligned to the *K. obovata* genome sequence using Exonerate v2.0 (https://www.ebi.ac.uk/Tools/psa/genewise/)^49^. Gene structures were generated using GeneWise v2.4.1 (ref. ^50^). Three ab initio prediction software programs, namely, Augustus v3.0.2 (http://bioinf.uni-greifswald.de/augustus/)^51^, Fgenesh (https://omictools.com/fgenesh-tool)^52^, and GlimmerHMM^53^, were employed for de novo gene prediction. Then, the homology-based and ab initio gene structures were merged into a nonredundant gene model using Maker v2.31.8 (ref. ^54^). TopHat v2.0.11 was used to map RNA-seq reads to the assembly^55^, and Cufflinks v2.2.1 (ref. ^56^) was applied to combine the mapping results for transcript structural predictions.

The protein sequences of the consensus gene set were aligned to seven protein databases, including GO (The Gene Ontology Consortium)^57^, KEGG (http://www.genome.jp/kegg/)^58^, InterPro (https://www.ebi.ac.uk/interpro/)^59^, Swiss-Prot (http://www.uniprot.org)^60^, and TrEMBL (http://www.uniprot.org/)^60^, for predicted gene annotation. The rRNAs were identified by aligning the rRNA template sequences from the Rfam^61^ database against the genome using the BLASTN algorithm with an E-value cutoff of 1E–5. The tRNAs were predicted using tRNAscan-SE v1.3.1 (http://lowelab.ucsc.edu/tRNAscan-SE/)^62^, and other ncRNAs were predicted by Infernal software (http://infernal.janelia.org/) against the Rfam database.

### Phylogenetic analysis

Genes from whole-genome sequences of ten species (*K. obovata*, *Amborella trichopoda*, *Arabidopsis thaliana*, *Dimocarpus longan*, *Morus notabilis*, *Populus trichocarpa*, *Rhizophora apiculata*, *Ricinus communis*, *Vitis vinifera*, and *Oryza sativa*) were used for gene-family clustering analysis. OrthoMCL v2.0.9 (ref. ^63^) was used to identify orthologous groups among the ten species. Pairwise similarities between all protein sequences were calculated using BLASTP with an *E*-value cutoff of 1E–5. To obtain reliable single-copy orthologous groups, we filtered out single-copy orthologous groups containing proteins of length <200 bp. MUSCLE v3.8.31 (ref. ^64^) was used to perform multisequence alignment of the protein sequences of the filtered single-copy orthologous group, and nucleotide alignment results were obtained by the corresponding relationship between protein sequences and nucleotide sequences. Finally, the nucleotide sequences of the single-copy orthologous group were connected to form a supergene, and then the data set was employed to construct a phylogenetic tree by using the GTR + gamma model in MrBayes^65^.

### Estimation of divergence time

The Markov chain Monte Carlo algorithm for Bayesian estimation was employed to infer the divergence time of each tree node using the MCMCTree module of PAML v4.7 (ref. ^66^). The nucleic acid replacement model used was the GTR model, and the molecular clock model used was the independent rate model. The MCMC process included 100,000 burn-in iterations and 1,000,000 sampling iterations (with a sample taken every 100 iterations). To obtain a more stable result, the same parameter was executed twice. Calibration times were obtained from TimeTree (http://www.timetree.org).

### Gene family expansion and contraction

We measured the expansion and contraction of orthologous gene families using CAFÉ 3 (https://github.com/hahnlab/CAFE)^14^. Based on maximum likelihood modeling of gene gain and loss, we analyzed gene families for signs of expansion or contraction using genomic data from the ten species.

### Collinearity analysis

Within collinear segments, genes are conserved in function and sequence and remain highly conserved during the evolution of species. We used the default parameters of JCVI v0.9.14 (https://pypi.org/project/jcvi/)^11^ to analyze the protein sequences of *K. obovata*, *R. apiculata*, and *V. vinifera* and obtained the gene pairs in collinear regions. Then, we used COGE (https://genomevolution.org/coge/) for online analysis, examined the relationship between *K*s peaks and collinear regions, and verified the WGD event experienced by the common ancestor of *K. obovata* and *R. apiculata*.

### Whole-genome duplication

We used *K*s distribution analysis to infer WGD events of *K. obovata* and *R. apiculata*. Diamond v0.9.24 (ref. ^67^) was used to conduct self-alignment of the protein sequences of the two species and then extract the mutual optimal alignment in the alignment results. Finally, Codeml in the PAML package was used to calculate the *K*s values^39,68^.

### MADS-box analysis

The hidden Markov model (HMM) profile of the MADS-box gene family (PF00319) was obtained from Pfam (http://pfam.xfam.org). MADS-box gene family proteins were separately searched with HMMER 3.1 (with the default parameters)^69^. InterProScan v 5.19 (ref. ^70^) was used to identify MADS-box gene family candidates in the genomes of *K. obovata* and *R. apiculata*. The genomic data of *R. apiculata* were downloaded from http://evolution.sysu.edu.cn/Sequences.html. MADS-box gene candidates were further confirmed with the 60 amino acid domains available from SMART^71^ and online BLAST analysis (https://www.ncbi.nlm.nih.gov). Specifically, the protein sequence set for the MADS-box gene candidates was subjected to BLAST analysis against the assembled transcriptomes of the roots, stems, leaves, flowers, and fruits of *K. obovata* with the TBLASTN program. A phylogenetic tree was then constructed using MEGA5 (ref. ^72^) with the default parameters.

### Disease resistance genes

Predicted proteins from the *K. obovata* and *R. apiculate* genomes were scanned using HMMER v3.1 (*E*-value cut-off of 1 × 10^−5^)^69^ using the HMM corresponding to the Pfam NLR protein family (NB-ARC: PF00931; TIR: PF01582; RPW8: PF05659; LRR: PF00560, PF07723, PF07725 and PF12799). To remove false-positive NB-ARC domain hits, InterProScan v5.19 was used to check the protein domains of the extracted sequences^70^. The NBS domains of the genes confirmed by both HMMER and InterProScan were extracted according to InterProScan annotation and aligned using MAFFT v7.310 (ref. ^63^); the alignment was then input into FastTree^73^ with the JTT model and visualized using EvolView^74^.

## Supplementary information

Chromosome-scale assembly of the *Kandelia obovata* genome

## Data Availability

Genome sequences have been submitted to the National Genomics Data Center (NGDC). PacBio whole-genome sequencing data and Illumina data have been deposited in BioProject/GSA (https://bigd.big.ac.cn/gsa.)^75^ under accession codes PRJCA002330/CRA002395 and the whole-genome assembly and annotation data have been deposited in BioProject/GWH (https://bigd.big.ac.cn/gwh)^76^ under accession codes PRJCA002330/GWHACBH00000000.
