# Supplementary material for: Chromosome-scale assembly of the Kandelia obovata genome
Source: Hortic Res. 2020 May 2;7:75. doi: 10.1038/s41438-020-0300-x (PMC7195387; doi:10.1038/s41438-020-0300-x)
Supplement: Supplementary file 1 — Chromosome-scale assembly of the Kandelia obovata genome [file 41438_2020_300_MOESM1_ESM.docx]

**Supplementary Information**

**Chromosome-scale assembly of the *Kandelia obovata* genome**

Min-Jie Hu^1*^, Wei-Hong Sun^2,3*^, Wen-Chieh Tsai^4^, Shuang Xiang^2,3^, Xing-Kai Lai^5^, De-Qiang Chen^2,3^, Xue-Die Liu^2^, Yi-Fan Wang^2^, Yi-Xun Le^2^, Si-Ming Chen^2,6^, Di-Yang Zhang^3^, Xia Yu^3^, Wen-Qi Hu^3^, Zhuang Zhou^3^, Yan-Qiong Chen^3^, Shuang-Quan Zou^2,3†^ and Zhong-Jian Liu^3†^

Running title: *Kandelia obovata* genome

**Content**

[Supplementary Figures 1](#_Toc34843960)

[Supplementary Figure 1. Genome size and heterozygosity estimation using 17 *K*-mer distribution. 1](#_Toc34843961)

[Supplementary Figure 2. Read length distribution of PacBio sequencing data. 2](#_Toc34843962)

[Supplementary Figure 3. Gene structure prediction statistical results. *K. obovata* compared with genetic elements of related species. 3](#_Toc34843963)

[Supplementary Figure 4. The sequence divergence rate of four different TEs using RepeatMasker annotation. 4](#_Toc34843964)

[Supplementary Figure 5. The sequence divergence rate of four different TEs using *de novo* annotation. 5](#_Toc34843965)

[Supplementary Figure 6. Venn diagram showing the gene function annotation results in NR, InterPro, KEGG, SwissProt and KOG. 6](#_Toc34843966)

[Supplementary Figure 7. Venn diagram shows the number of orthologous gene families in *K. obovata*, *R. apiculata*, *R. communis*, and *P. trichocarpa*. 7](#_Toc34843967)

[Supplementary Figure 8. Orthologous genes in *K. obovata* and other species. 8](#_Toc34843968)

[Supplementary Figure 9. Phylogenetic relationships and divergence times between *K. obovata* and other plant species. 9](#_Toc34843969)

[Supplementary Figure 10. The map of *K*s distribution. 10](#_Toc34843970)

[Supplementary Tables 11](#_Toc34843971)

[Supplementary Table 1. The statistics of sequencing raw data from the Pacific Biosciences RS II sequencing platform. 11](#_Toc34843972)

[Supplementary Table 2. Chromosome length by Hi-C assembly. 12](#_Toc34843973)

[Supplementary Table 3. The prediction of gene structures of the *K. obovata*. 13](#_Toc34843974)

[Supplementary Table 4. The number of protein coding genes supported by *de novo*, transcriptome data and homology prediction. 14](#_Toc34843975)

[Supplementary Table 5. BUSCO assessment of the *K. obovata* genome 15](#_Toc34843976)

[Supplementary Table 6. Statistics on the annotation of non-coding RNA of the *K. obovata* genome. 16](#_Toc34843977)

[Supplementary Table 7. The statistical results of repeat sequences. 1](#_Toc34843978)

[Supplementary Table 8. Statistics of repeat sequences in *K. obovata*. 2](#_Toc34843979)

[Supplementary Table 9. The statistical results of functional annotation. 3](#_Toc34843980)

[Supplementary Table 10. Statistical results of clustered gene families. 4](#_Toc34843981)

[Supplementary Table 11. Expanded genes families in GO terms. 5](#_Toc34843982)

[Supplementary Table 12. Contracted genes families in GO terms. 7](#_Toc34843983)

[Supplementary Table 13. The results of whole genome collinearity analysis. 23](#_Toc34843984)

[Supplementary Table 14. List of MADS-box genes identified in *K. obovata* and *R. apiculata*. 24](#_Toc34843985)

[Supplementary Table 15. List of reference R genes from different species. 28](#_Toc34843986)

# Supplementary Figures


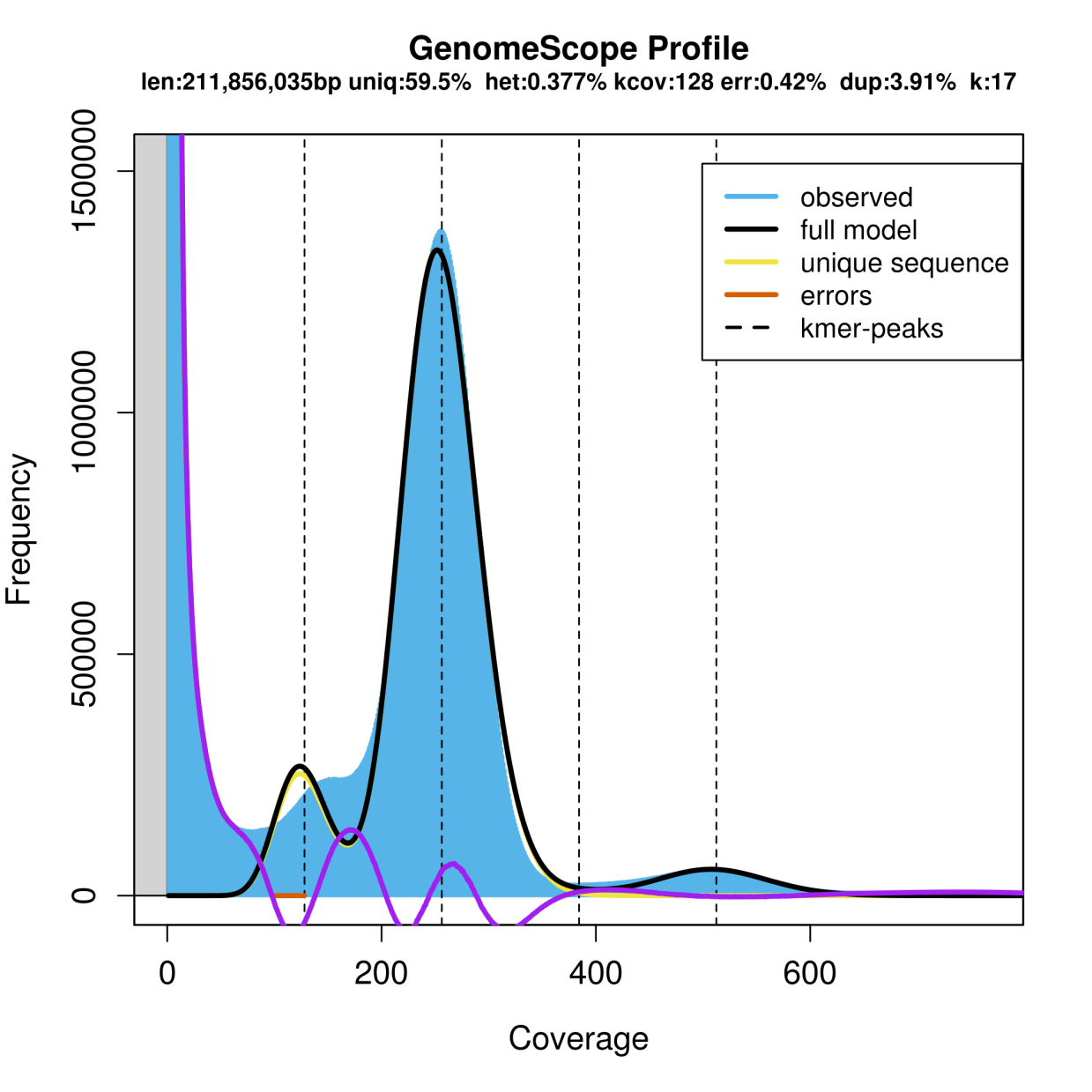


## Supplementary Figure 1. Genome size and heterozygosity estimation using 17 *K*-mer distribution.


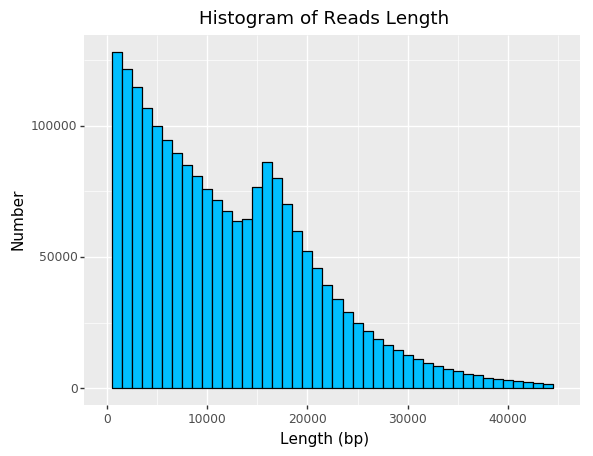


## Supplementary Figure 2. Read length distribution of PacBio sequencing data.

**
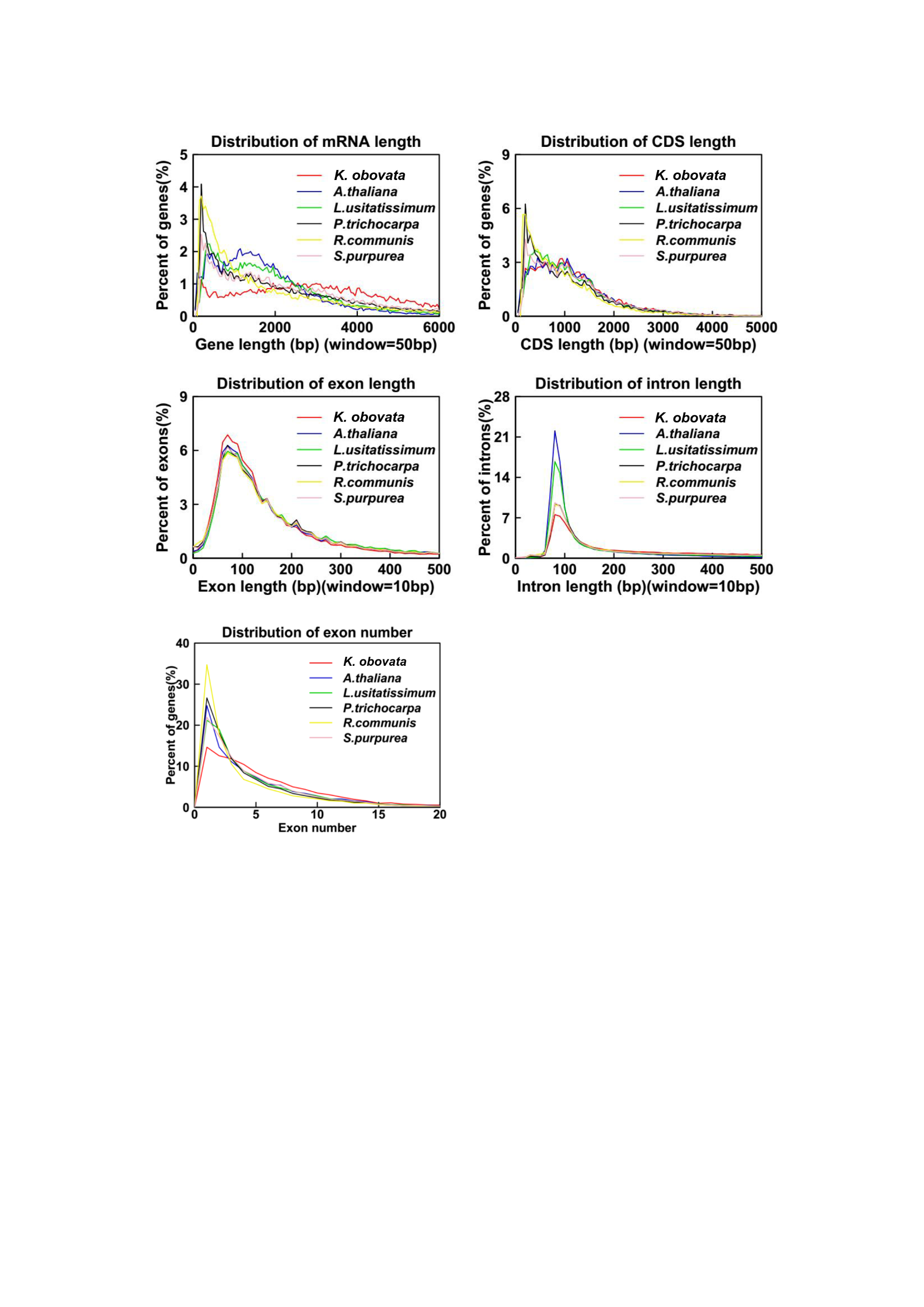
**

## Supplementary Figure 3. Gene structure prediction statistical results. *K. obovata* compared with genetic elements of related species.

Window refers to the length represented by each point on the horizonal coordinate.

**
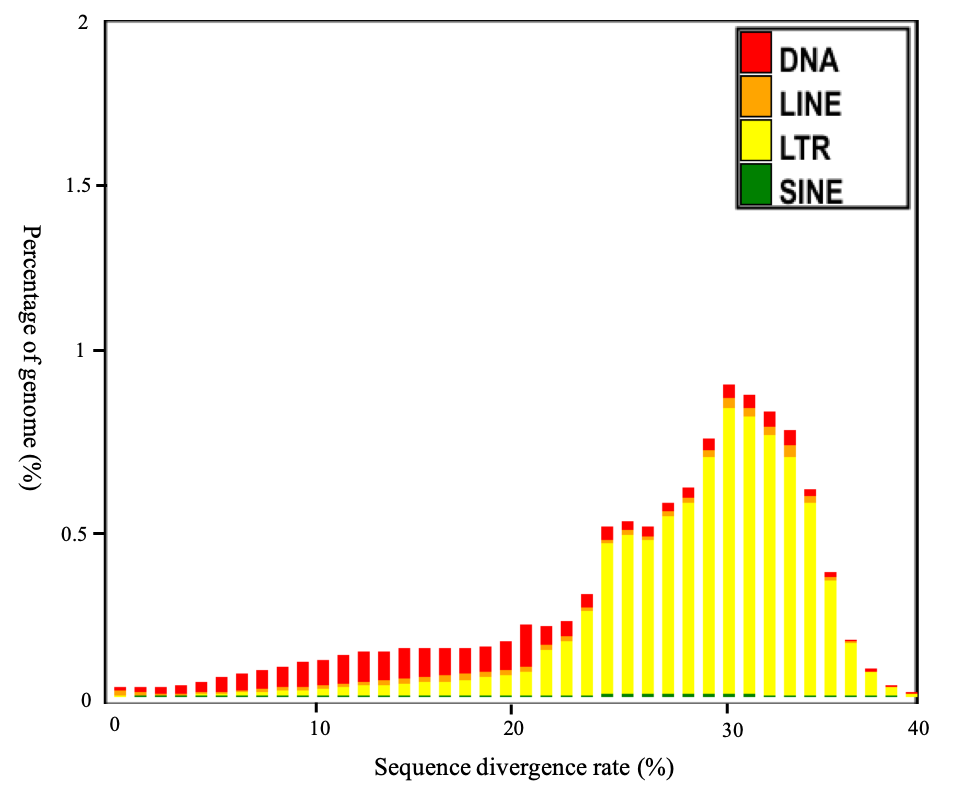
**

## Supplementary Figure 4. The sequence divergence rate of four different TEs using RepeatMasker annotation.

**
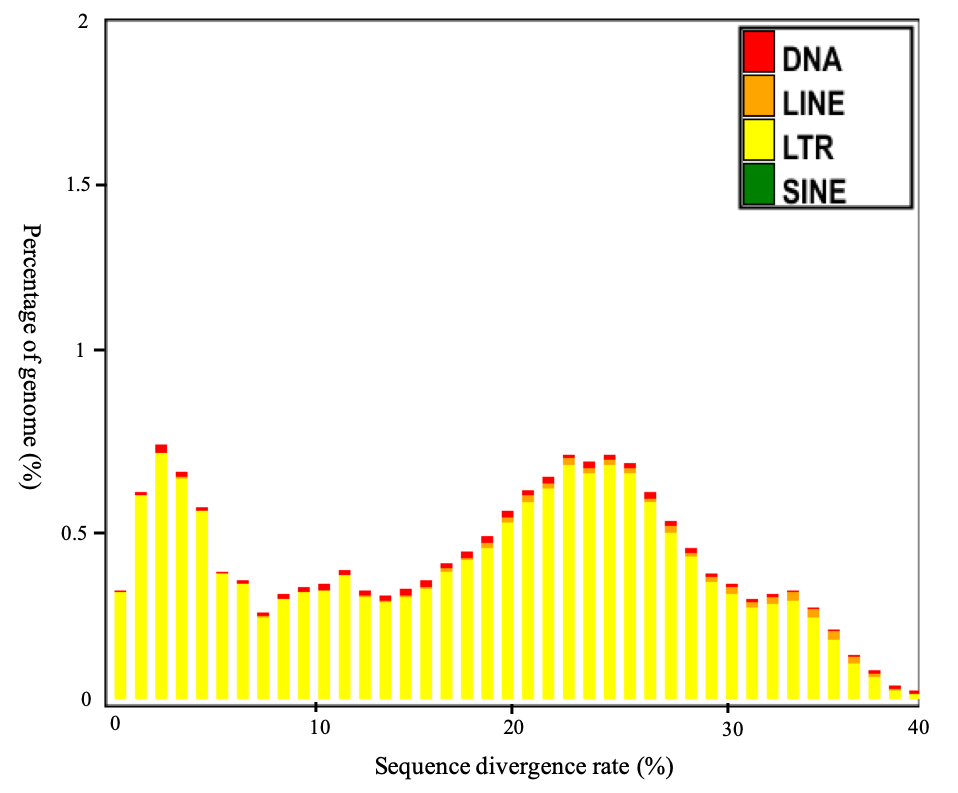
**

## Supplementary Figure 5. The sequence divergence rate of four different TEs using *de novo* annotation.


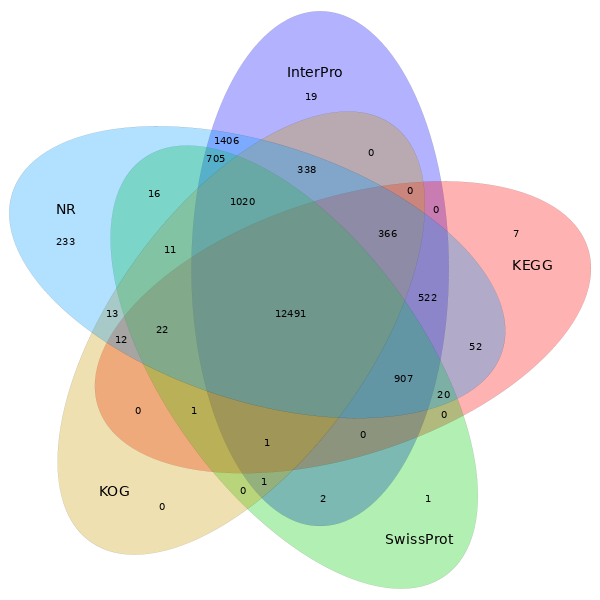


## Supplementary Figure 6. Venn diagram showing the gene function annotation results in NR, InterPro, KEGG, SwissProt and KOG.


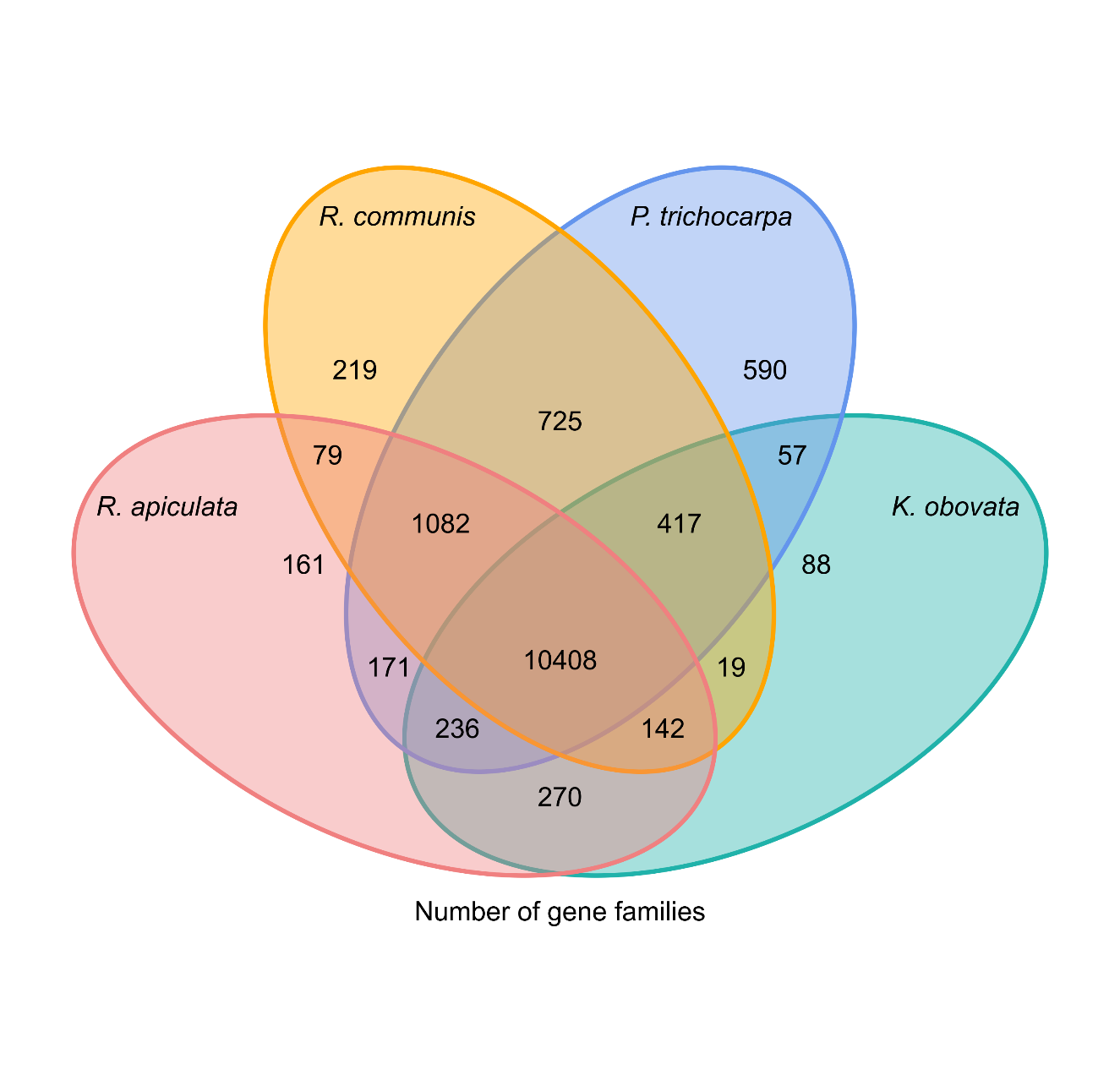


## Supplementary Figure 7. Venn diagram shows the number of orthologous gene families in *K. obovata*, *R. apiculata*, *R. communis*, and *P. trichocarpa*.

**
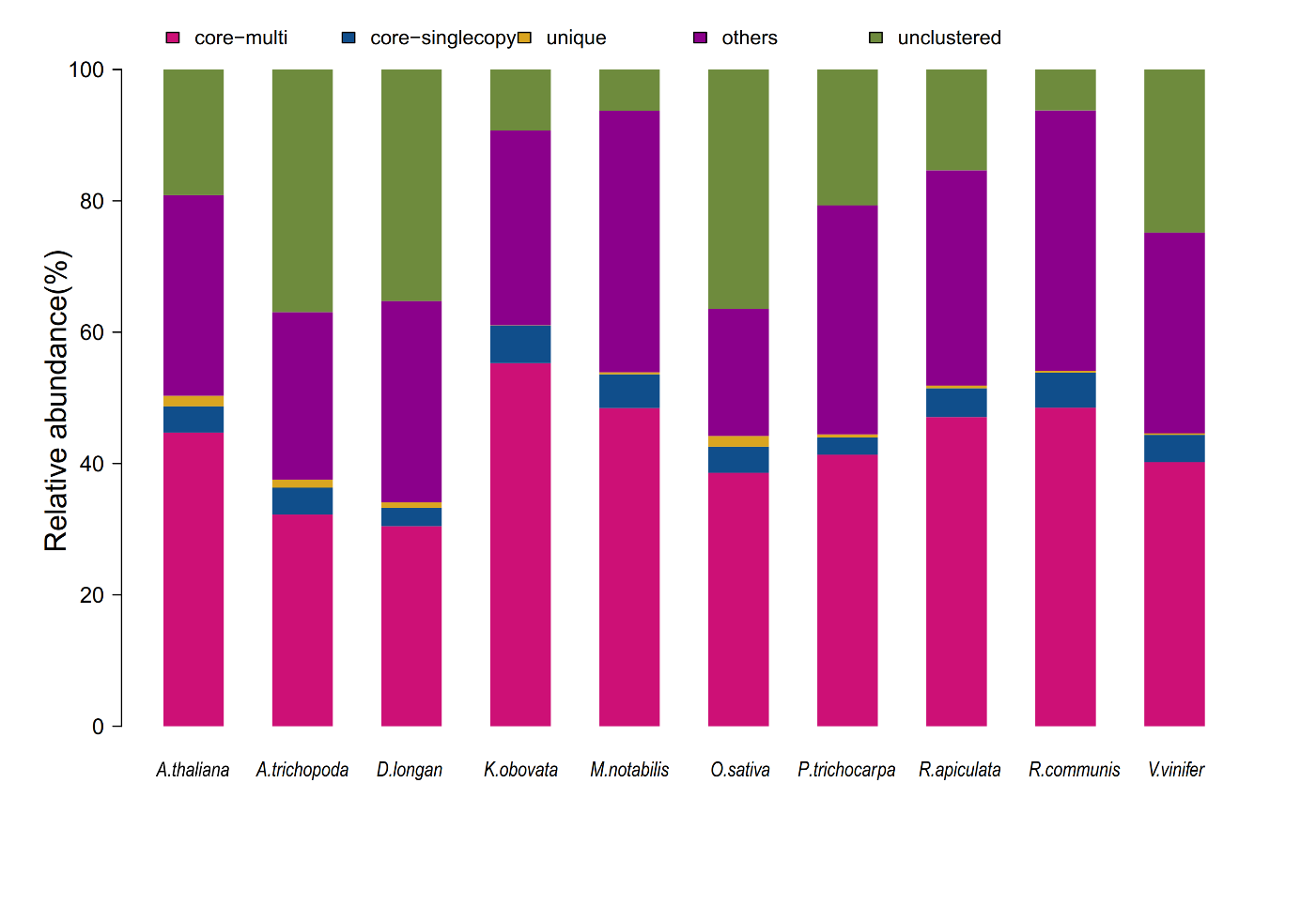
**

## Supplementary Figure 8. Orthologous genes in *K. obovata* and other species.


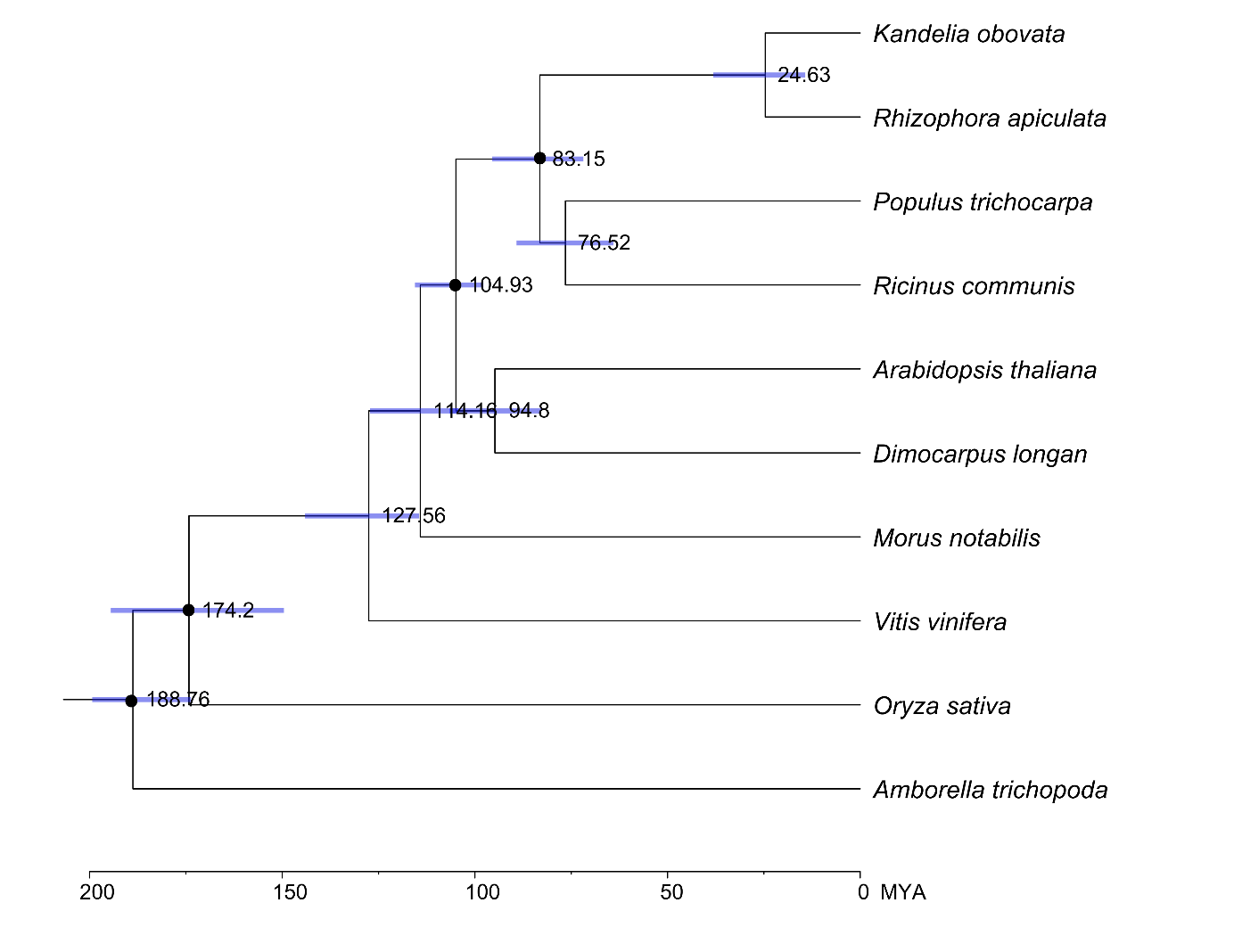


## Supplementary Figure 9. Phylogenetic relationships and divergence times between *K. obovata* and other plant species.


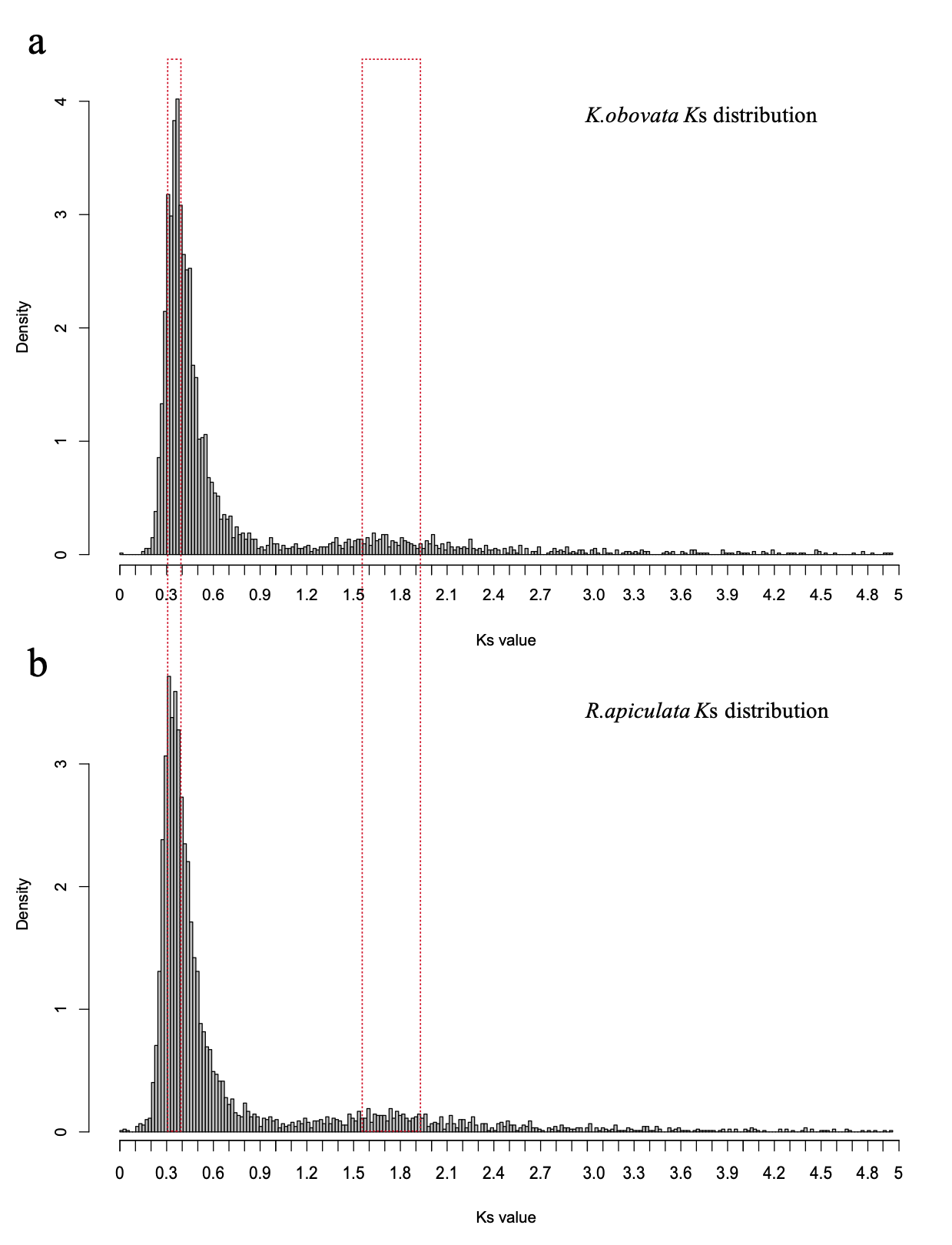


## Supplementary Figure 10. The map of *K*s distribution.

a. *K*s distribution of *K. obovata*. b. *K*s distribution of *R. apiculata*.

# Supplementary Tables

## Supplementary Table 1. The statistics of sequencing raw data from the Pacific Biosciences RS II sequencing platform.

| **Illumina sequence** | **Insert size (bp)** | **Read Length (bp)** | **Raw data (Gb)** | **Clean data (Gb)** |
| --- | --- | --- | --- | --- |
|  | **500** | **150** | **76.86** | **65.27** |

| **PacBio sequence** | ID | **ZMNUM** | **Total Bases**  **(Gb)** | **Total reads** | **Average**  **length (bp)** | **Max**  **length (bp)** | **N50 length (bp)** |
| --- | --- | --- | --- | --- | --- | --- | --- |
|  | B01 | 780795 | 11.46 | 1,072,434 | 10,687.01 | 83,096 | 16,505 |
|  | A01 | 719150 | 13.54 | 1,022,792 | 13,239.36 | 91,546 | 18,738 |
|  | Total | **-** | 25.00 | 2,095,226 | 11,932.95 | 91,546 | 17,621 |

## Supplementary Table 2. Chromosome length by Hi-C assembly.

| **Chromosome ID** |  | **Length(bp)** |
| --- | --- | --- |
| Chr01 |  | 13,797,742 |
| Chr02 |  | 13,452,090 |
| Chr03 |  | 13,118,466 |
| Chr04 |  | 12,048,136 |
| Chr05 |  | 11,637,810 |
| Chr06 |  | 10,559,637 |
| Chr07 |  | 10,067,174 |
| Chr08 |  | 10,026,007 |
| Chr09 |  | 9,869,847 |
| Chr10 |  | 9,843,429 |
| Chr11 |  | 9,799,643 |
| Chr12 |  | 9,654,243 |
| Chr13 |  | 8,475,962 |
| Chr14 |  | 8,171,194 |
| Chr15 |  | 7,574,532 |
| Chr16 |  | 7,500,541 |
| Chr17 |  | 7,389,461 |
| Chr18 |  | 5,028,210 |

## Supplementary Table 3. The prediction of gene structures of the *K. obovata*.

|  | **Gene set** | **Number** | **Average mRNA length (bp)** | **Average mRNA length (bp)** | **Average mRNA length (bp)** | **Average CDS length (bp)** | **Average exon per gene** | **Average**  **Exon**  **Length (bp)** | **Average**  **intron**  **length (bp)** |
| --- | --- | --- | --- | --- | --- | --- | --- | --- | --- |
| **De novo** | **Augustus** | 21,809 | 3325.64 | 3325.64 | 3325.64 | 1355.98 | 5.94 | 228.38 | 398.93 |
|  | **SNAP** | 34,046 | 2723.21 | 2723.21 | 2723.21 | 880.34 | 4.63 | 190.27 | 508.12 |
| **Homolog** | ***Arabidopsis thaliana*** | 64,158 | 2172.95 | 2172.95 | 941.39 | 941.39 | 3.78 | 248.83 | 442.47 |
|  | ***Linum usitatissimum*** | 122,215 | 1991.40 | 1991.40 | 854.14 | 854.14 | 3.44 | 248.05 | 465.45 |
|  | ***Populus trichocarpa*** | 127,059 | 2108.49 | 2108.49 | 890.80 | 890.80 | 3.36 | 265.37 | 516.66 |
|  | ***Ricinus communis*** | 68,261 | 2033.65 | 2033.65 | 889.19 | 889.19 | 3.51 | 253.48 | 456.34 |
|  | ***Salix purpurea*** | 118,208 | 2125.57 | 2125.57 | 912.14 | 912.14 | 3.48 | 262.10 | 489.26 |
| **Transcriptome** | **Kc_Fl** | 68,453 | 3076.05 | 3076.05 | 1431.28 | 1431.28 | 4.84 | 295.46 | 427.85 |
|  | **Kc_Ro** | 82,212 | 3168.27 | 3168.27 | 1511.05 | 1511.05 | 4.85 | 311.33 | 430.06 |
|  | **Kc_St** | 70,320 | 2636.95 | 2636.95 | 1198.17 | 1198.17 | 4.24 | 282.54 | 443.98 |
|  | **Kc_Le** | 92,849 | 3233.67 | 3233.67 | 1571.39 | 1571.39 | 4.99 | 314.81 | 416.44 |
|  | **Kc_Fr I** | 71,857 | 3248.26 | 3248.26 | 1536.14 | 1536.14 | 4.99 | 307.57 | 428.63 |
|  | **Kc_Fr II** | 85,292 | 3298.91 | 3298.91 | 1555.19 | 1555.19 | 5.09 | 305.68 | 426.59 |
|  | **Kc_Fr III** | 82,364 | 3329.58 | 3329.58 | 1614.87 | 1614.87 | 5.24 | 308.33 | 404.66 |
|  | **Kc_Fr IV** | 71,438 | 3306.99 | 3306.99 | 1581.25 | 1581.25 | 5.10 | 310.11 | 421.02 |
| **Maker** | | 19,138 | 3893.59 | 3893.59 | 1291.12 | 1291.12 | 6.09 | 212.12 | 378.99 |

## Supplementary Table 4. The number of protein coding genes supported by *de novo*, transcriptome data and homology prediction.

|  | **>=30% overlap** | | **>=50% overlap** | | **>=80% overlap** | |
| --- | --- | --- | --- | --- | --- | --- |
|  | **Number** | **Ration (%)** | **Number** | **Ration (%)** | **Number** | **Ration (%)** |
| **R(single)** | 40 | 0.21 | 52 | 0.27 | 145 | 0.76 |
| **R(more)** | 163 | 0.85 | 212 | 1.11 | 662 | 3.46 |
| **H(single)** | 0 | 0.00 | 0 | 0.00 | 2 | 0.01 |
| **H(more)** | 0 | 0.00 | 0 | 0.00 | 7 | 0.037 |
| **P(single)** | 16 | 0.085 | 77 | 0.40 | 298 | 3.29 |
| **P(more)** | 17 | 0.0089 | 47 | 0.25 | 146 | 1.56 |
| **HR** | 73 | 0.38 | 114 | 0.60 | 481 | 2.51 |
| **PR** | 1,652 | 8.63 | 1,899 | 9.92 | 3,676 | 19.21 |
| **PH** | 93 | 0.49 | 184 | 0.96 | 407 | 2.13 |
| **PHR** | 17,082 | 89.26 | 16,539 | 84.42 | 13,153 | 68.73 |
| **Total** | 19,136 | 99.99 | 19,124 | 99.93 | 18,980 | 99.17 |

**R:** represents the number of genes supported by transcriptome data; **P:** represents the number of genes supported by the results of *de novo* prediction; **H:** represents the number of genes supported by the homology prediction. **Single** means that there is only one data evidence to support. **More** means that there is much supporting data evidence. **Overlap:** represents the ration of the final gene set to the overlap of the CDS region of various predicted results.

## Supplementary Table 5. BUSCO assessment of the *K. obovata* genome

| **Type** | **Match percentage (%)** | **Number** | **Percentage** |
| --- | --- | --- | --- |
| Complete | Complete and single-copy BUSCOs (S) | 1,204 | 87.6% |
|  | Complete and duplicated BUSCOs (D) | 33 | 2.4% |
|  | Fragmented BUSCOs (F) | 60 | 4.4% |
|  | Missing BUSCOs (M) | 78 | 5.6% |
|  | Total BUSCO groups searched | 1,375 | - |

## Supplementary Table 6. Statistics on the annotation of non-coding RNA of the *K. obovata* genome.

|  | **Type** | **Copy** | **Average length (bp)** | **Total length (bp)** | **% of genome** |
| --- | --- | --- | --- | --- | --- |
|  | **miRNA** | 105 | 129.59 | 13,607 | 0.0076 |
|  | **tRNA** | 307 | 75 | 23,025 | 0.013 |
|  | **rRNA** | 167 | 239.84 | 40,053 | 0.023 |
|  | **18S** | 65 | 426.38 | 27,715 | 0.016 |
|  | **28S** | 29 | 127.10 | 3,686 | 0.0021 |
| **rRNA** | **5.8S** | 14 | 144.79 | 2,027 | 0.0011 |
|  | **5S** | 59 | 112.29 | 6,625 | 0.0037 |
|  | **snRNA** | 199 | 110.45 | 21,979 | 0.012 |
|  | **CD-box** | 126 | 95.42 | 12,023 | 0.0068 |
| **snRNA** | **HACA-box** | 34 | 127.94 | 4,350 | 0.0024 |
|  | **splicing** | 39 | 143.74 | 5,606 | 0.0032 |

## Supplementary Table 7. The statistical results of repeat sequences.

| **Type** | **Repeat Size (bp)** | **% of genome** |
| --- | --- | --- |
| **TRF** | 3,389,914 | 1.90 |
| **RepeatMasker** | 18,296,031 | 10.28 |
| **RepeatProteinMask** | 14,359,412 | 8.07 |
| **De novo** | 37,424,174 | 21.03 |
| **Total** | 42,837,321 | 24.07 |

## Supplementary Table 8. Statistics of repeat sequences in *K. obovata*.

|  | **RepBase TEs** | | **TE Proteins** | | **De novo** | | **Combined TEs** | |
| --- | --- | --- | --- | --- | --- | --- | --- | --- |
| **Length**  **(bp)** | **% in Genome** | **Length (bp)** | **% in Genome** | **Length**  **(bp)** | **% in Genome** | **Length**  **(bp)** | **% in Genome** |  |
| **DNA** | 2,490,849 | 1.40 | 516,776 | 0.29 | 639,267 | 0.36 | 3,186,890 | 1.79 |
| **LINE** | 915,761 | 0.51 | 816,037 | 0.46 | 673,931 | 0.38 | 1,757,602 | 0.99 |
| **LTR** | 15,200,009 | 8.54 | 13,032,873 | 7.32 | 29,062,593 | 16.33 | 30,850,798 | 17.33 |
| **SINE** | 162,812 | 0.091 | 0 | 0 | 0 | 0 | 162,812 | 0.091 |
| **Other** | 3757 | 0.0021 | 0 | 0 | 0 | 0 | 3,757 | 0.0021 |
| **Unknown** | 0 | 0 | 0 | 0 | 8,014,484 | 4.50 | 8,014,484 | 4.50 |
| **Total** | 18,296,031 | 10.28 | 14,359,412 | 8.07 | 37,328,782 | 20.97 | 41,388,057 | 23.25 |

*LINE, long interspersed nuclear element; SINE, short interspersed element; LTR, long terminal repeat;

*Denovo+Repbase denotes transposable elements identified by RepeatMasker (<http://www.repeatmasker.org>) with default options after RepeatModeler/RepeatScout/Piler/LTR_finder software use with RepBase database prediction.

*TE proteins were transposable elements identified in the genome through the annotation of Repeat ProteinMask software using the RepBase database.

*Combined TEs involved a combination of the above two methods.

*Unknown repeat sequences could not be clustered by Repeat Masker.

## Supplementary Table 9. The statistical results of functional annotation.

| **Values** | **Total** | **Nr** | **Swissprot** | **KEGG** | **KOG** | **TrEMBL** | **Interpro** | **GO** | **Overall** |
| --- | --- | --- | --- | --- | --- | --- | --- | --- | --- |
| **Number** | 19,138 | 18,134 | 15,198 | 14,401 | 14,276 | 18,229 | 17,778 | 11,124 | 18,268 |
| **Percentage** | **-** | 94.75% | 79.41% | 75.25% | 74.60% | 95.25% | 92.89% | 58.13% | 95.45% |

## Supplementary Table 10. Statistical results of clustered gene families.

| **Species** | **Genes** | **Unclustered genes** | **Clustered genes** | **Families** | **Unique families** | **Unique families**  **genes** | **Common families** | **Common**  **families**  **genes** | **Single copy** | **Average genes per**  **family** |
| --- | --- | --- | --- | --- | --- | --- | --- | --- | --- | --- |
| ***A. thaliana*** | 27416 | 5245 | 22171 | 12221 | 55 | 442 | 7237 | 13352 | 1095 | 1.814 |
| ***A. trichopoda*** | 26846 | 9919 | 16927 | 12024 | 56 | 327 | 7237 | 9750 | 1095 | 1.408 |
| ***D. longan*** | 39282 | 13851 | 25431 | 13142 | 55 | 335 | 7237 | 13061 | 1095 | 1.935 |
| ***K. obovata*** | 19138 | 1774 | 17364 | 11637 | 3 | 8 | 7237 | 11678 | 1095 | 1.492 |
| ***M. notabilis*** | 21348 | 1338 | 20010 | 12748 | 17 | 69 | 7237 | 11437 | 1095 | 1.57 |
| ***O. sativa*** | 27694 | 10093 | 17601 | 10569 | 75 | 453 | 7237 | 11783 | 1095 | 1.665 |
| ***P.s trichocarpa*** | 41335 | 8550 | 32785 | 13686 | 39 | 188 | 7237 | 18180 | 1095 | 2.396 |
| ***R. apiculata*** | 24911 | 3826 | 21085 | 12549 | 7 | 107 | 7237 | 12817 | 1095 | 1.68 |
| ***R. communis*** | 20558 | 1280 | 19278 | 13091 | 8 | 62 | 7237 | 11065 | 1095 | 1.473 |
| ***V. vinifera*** | 26346 | 6543 | 19803 | 12462 | 18 | 65 | 7237 | 11685 | 1095 | 1.589 |

## Supplementary Table 11. Expanded genes families in GO terms.

| **Gene ID** | **GO** |
| --- | --- |
| **Maker00000324** | NA |
| **Maker00003297** | biological_process:GO:0007017//microtubule-based process;cellular_component:GO:0005874//microtubule;GO:0005737//cytoplasm;molecular_function:GO:0005200//structural constituent of cytoskeleton;GO:0005525//GTP binding;GO:0003924//GTPase activity; |
| **Maker00012246** | NA |
| **Maker00014359** | NA |
| **Maker00014414** | NA |
| **Maker00016046** | NA |
| **Maker00017852** | NA |
| **Maker00018329** | NA |
| **Maker00018512** | biological_process:GO:0007017//microtubule-based process;cellular_component:GO:0005874//microtubule;GO:0005737//cytoplasm;molecular_function:GO:0005200//structural constituent of cytoskeleton;GO:0005525//GTP binding;GO:0003924//GTPase activity; |
| **Maker00002094** | biological_process:GO:0006412//translation;cellular_component:GO:0015934//large ribosomal subunit;molecular_function:GO:0003723//RNA binding;GO:0003735//structural constituent of ribosome; |
| **Maker00006776** | biological_process:GO:0006412//translation;cellular_component:GO:0015934//large ribosomal subunit;molecular_function:GO:0003723//RNA binding;GO:0003735//structural constituent of ribosome; |
| **Maker00007512** | biological_process:GO:0006412//translation;cellular_component:GO:0015934//large ribosomal subunit;molecular_function:GO:0003723//RNA binding;GO:0003735//structural constituent of ribosome; |
| **Maker00008281** | biological_process:GO:0006412//translation;GO:0002181//cytoplasmic translation;cellular_component:GO:0015934//large ribosomal subunit;GO:0022625//cytosolic large ribosomal subunit;molecular_function:GO:0003723//RNA binding;GO:0003735//structural constituent of ribosome; |
| **Maker00012421** | biological_process:GO:0006412//translation;cellular_component:GO:0015934//large ribosomal subunit;molecular_function:GO:0003723//RNA binding;GO:0003735//structural constituent of ribosome; |
| **Maker00000992** | NA |
| **Maker00003877** | NA |
| **Maker00004228** | NA |
| **Maker00015023** | NA |
| **Maker00012225** | biological_process:GO:0007017//microtubule-based process;cellular_component:GO:0005874//microtubule;GO:0005737//cytoplasm;molecular_function:GO:0005200//structural constituent of cytoskeleton;GO:0005525//GTP binding;GO:0003924//GTPase activity; |
| **Maker00016025** | NA |
| **Maker00018077** | NA |
| **Maker00018595** | NA |

## Supplementary Table 12. Contracted genes families in GO terms.

| **Gene ID** | **GO** |
| --- | --- |
| **Maker00000575** | molecular_function:GO:0005524//ATP binding;GO:0004672//protein kinase activity;GO:0030247//polysaccharide binding; |
| **Maker00001797** | NA |
| **Maker00003065** | NA |
| **Maker00004297** | cellular_component:GO:0016021//integral component of membrane;molecular_function:GO:0005524//ATP binding;GO:0004672//protein kinase activity;GO:0030247//polysaccharide binding; |
| **Maker00008654** | cellular_component:GO:0016021//integral component of membrane;molecular_function:GO:0005524//ATP binding;GO:0004672//protein kinase activity; |
| **Maker00009012** | cellular_component:GO:0016021//integral component of membrane;molecular_function:GO:0005524//ATP binding;GO:0004672//protein kinase activity; |
| **Maker00008903** | biological_process:GO:0051762//sesquiterpene biosynthetic process;molecular_function:GO:0010334//sesquiterpene synthase activity;GO:0000287//magnesium ion binding; |
| **Maker00009687** | biological_process:GO:0008152//metabolic process;molecular_function:GO:0000287//magnesium ion binding;GO:0010333//terpene synthase activity; |
| **Maker00013197** | biological_process:GO:1901928//cadinene biosynthetic process;molecular_function:GO:0000287//magnesium ion binding;GO:0047461//(+)-delta-cadinene synthase activity;GO:0102877//alpha-copaene synthase activity; |
| **Maker00013275** | biological_process:GO:1901928//cadinene biosynthetic process;molecular_function:GO:0000287//magnesium ion binding;GO:0047461//(+)-delta-cadinene synthase activity;GO:0102877//alpha-copaene synthase activity; |
| **Maker00013825** | biological_process:GO:1901928//cadinene biosynthetic process;molecular_function:GO:0000287//magnesium ion binding;GO:0047461//(+)-delta-cadinene synthase activity;GO:0102877//alpha-copaene synthase activity; |
| **Maker00013839** | biological_process:GO:1901928//cadinene biosynthetic process;molecular_function:GO:0000287//magnesium ion binding;GO:0047461//(+)-delta-cadinene synthase activity;GO:0102877//alpha-copaene synthase activity; |
| **Maker00000903** | biological_process:GO:0044550//secondary metabolite biosynthetic process;cellular_component:GO:0016020//membrane;GO:0016021//integral component of membrane;molecular_function:GO:0016709//oxidoreductase activity, acting on paired donors, with incorporation or reduction of molecular oxygen, NAD(P)H as one donor, and incorporation of one atom of oxygen;GO:0020037//heme binding;GO:0005506//iron ion binding; |
| **Maker00001091** | biological_process:GO:0044550//secondary metabolite biosynthetic process;cellular_component:GO:0016020//membrane;GO:0016021//integral component of membrane;molecular_function:GO:0016709//oxidoreductase activity, acting on paired donors, with incorporation or reduction of molecular oxygen, NAD(P)H as one donor, and incorporation of one atom of oxygen;GO:0020037//heme binding;GO:0005506//iron ion binding;GO:0016705//oxidoreductase activity, acting on paired donors, with incorporation or reduction of molecular oxygen;GO:0004497//monooxygenase activity; |
| **Maker00002826** | cellular_component:GO:0016021//integral component of membrane;molecular_function:GO:0016705//oxidoreductase activity, acting on paired donors, with incorporation or reduction of molecular oxygen;GO:0020037//heme binding;GO:0005506//iron ion binding;GO:0004497//monooxygenase activity; |
| **Maker00004494** | molecular_function:GO:0047085//hydroxyphenylacetonitrile 2-monooxygenase activity;GO:0020037//heme binding;GO:0005506//iron ion binding;GO:0050592//4-hydroxyphenylacetaldehyde oxime monooxygenase activity; |
| **Maker00006176** | NA |
| **Maker00008747** | cellular_component:GO:0016021//integral component of membrane;molecular_function:GO:0016705//oxidoreductase activity, acting on paired donors, with incorporation or reduction of molecular oxygen;GO:0020037//heme binding;GO:0005506//iron ion binding;GO:0004497//monooxygenase activity; |
| **Maker00011824** | NA |
| **Maker00018012** | NA |
| **Maker00006945** | molecular_function:GO:0016614//oxidoreductase activity, acting on CH-OH group of donors;GO:0050660//flavin adenine dinucleotide binding; |
| **Maker00012194** | NA |
| **Maker00012311** | molecular_function:GO:0016614//oxidoreductase activity, acting on CH-OH group of donors;GO:0050660//flavin adenine dinucleotide binding; |
| **Maker00012343** | molecular_function:GO:0016614//oxidoreductase activity, acting on CH-OH group of donors;GO:0050468//reticuline oxidase activity;GO:0050660//flavin adenine dinucleotide binding; |
| **Maker00001353** | NA |
| **Maker00006112** | NA |
| **Maker00018555** | cellular_component:GO:0016021//integral component of membrane; |
| **Maker00018737** | NA |
| **Maker00018856** | NA |
| **Maker00018966** | NA |
| **Maker00019070** | cellular_component:GO:0016021//integral component of membrane;molecular_function:GO:0016301//kinase activity; |
| **Maker00011283** | cellular_component:GO:0005576//extracellular region;molecular_function:GO:0045735//nutrient reservoir activity;GO:0030145//manganese ion binding; |
| **Maker00011287** | NA |
| **Maker00011406** | cellular_component:GO:0005576//extracellular region;molecular_function:GO:0045735//nutrient reservoir activity;GO:0030145//manganese ion binding; |
| **Maker00011448** | cellular_component:GO:0005576//extracellular region;molecular_function:GO:0045735//nutrient reservoir activity;GO:0030145//manganese ion binding; |
| **Maker00011467** | cellular_component:GO:0005576//extracellular region;molecular_function:GO:0045735//nutrient reservoir activity;GO:0030145//manganese ion binding; |
| **Maker00011483** | cellular_component:GO:0005576//extracellular region;molecular_function:GO:0045735//nutrient reservoir activity;GO:0030145//manganese ion binding; |
| **Maker00011493** | NA |
| **Maker00012224** | cellular_component:GO:0005618//cell wall;GO:0005576//extracellular region;molecular_function:GO:0045735//nutrient reservoir activity;GO:0030145//manganese ion binding; |
| **Maker00012344** | cellular_component:GO:0005618//cell wall;GO:0005576//extracellular region;molecular_function:GO:0004784//superoxide dismutase activity;GO:0045735//nutrient reservoir activity;GO:0030145//manganese ion binding; |
| **Maker00012366** | cellular_component:GO:0005618//cell wall;GO:0005576//extracellular region;molecular_function:GO:0004784//superoxide dismutase activity;GO:0045735//nutrient reservoir activity;GO:0030145//manganese ion binding; |
| **Maker00004694** | biological_process:GO:0006468//protein phosphorylation;cellular_component:GO:0016021//integral component of membrane;GO:0005886//plasma membrane;molecular_function:GO:0004674//protein serine/threonine kinase activity;GO:0005524//ATP binding; |
| **Maker00004726** | biological_process:GO:0006468//protein phosphorylation;cellular_component:GO:0016021//integral component of membrane;GO:0005886//plasma membrane;molecular_function:GO:0004674//protein serine/threonine kinase activity;GO:0005524//ATP binding;GO:0004672//protein kinase activity; |
| **Maker00015550** | cellular_component:GO:0016021//integral component of membrane;molecular_function:GO:0004674//protein serine/threonine kinase activity;GO:0005524//ATP binding; |
| **Maker00015635** | cellular_component:GO:0016021//integral component of membrane;molecular_function:GO:0004674//protein serine/threonine kinase activity;GO:0005524//ATP binding; |
| **Maker00017379** | biological_process:GO:0006468//protein phosphorylation;cellular_component:GO:0016021//integral component of membrane;GO:0005886//plasma membrane;molecular_function:GO:0004674//protein serine/threonine kinase activity;GO:0005524//ATP binding;GO:0004672//protein kinase activity; |
| **Maker00019068** | NA |
| **Maker00011514** | NA |
| **Maker00011997** | biological_process:GO:0007165//signal transduction;cellular_component:GO:0016020//membrane;GO:0016021//integral component of membrane; |
| **Maker00013793** | cellular_component:GO:0016021//integral component of membrane; |
| **Maker00018964** | cellular_component:GO:0016021//integral component of membrane; |
| **Maker00018993** | cellular_component:GO:0016021//integral component of membrane; |
| **Maker00002590** | NA |
| **Maker00006073** | NA |
| **Maker00011042** | NA |
| **Maker00011322** | biological_process:GO:0006952//defense response;molecular_function:GO:0043531//ADP binding; |
| **Maker00013616** | NA |
| **Maker00015861** | NA |
| **Maker00012971** | biological_process:GO:0006952//defense response;molecular_function:GO:0043531//ADP binding; |
| **Maker00004016** | NA |
| **Maker00004152** | cellular_component:GO:0016021//integral component of membrane;molecular_function:GO:0004970//ionotropic glutamate receptor activity; |
| **Maker00004183** | cellular_component:GO:0016021//integral component of membrane;molecular_function:GO:0004970//ionotropic glutamate receptor activity; |
| **Maker00009937** | NA |
| **Maker00002287** | biological_process:GO:0006749//glutathione metabolic process;GO:0009407//toxin catabolic process;cellular_component:GO:0005737//cytoplasm;molecular_function:GO:0004364//glutathione transferase activity;GO:0016740//transferase activity; |
| **Maker00007878** | biological_process:GO:0006749//glutathione metabolic process;GO:0009407//toxin catabolic process;cellular_component:GO:0005737//cytoplasm;molecular_function:GO:0004364//glutathione transferase activity; |
| **Maker00017976** | molecular_function:GO:0004462//lactoylglutathione lyase activity;GO:0016740//transferase activity; |
| **Maker00017991** | NA |
| **Maker00001169** | molecular_function:GO:0008146//sulfotransferase activity; |
| **Maker00001415** | molecular_function:GO:0008146//sulfotransferase activity; |
| **Maker00003486** | molecular_function:GO:0008146//sulfotransferase activity; |
| **Maker00003670** | molecular_function:GO:0008146//sulfotransferase activity; |
| **Maker00006654** | molecular_function:GO:0008146//sulfotransferase activity; |
| **Maker00010086** | molecular_function:GO:0008146//sulfotransferase activity; |
| **Maker00012417** | NA |
| **Maker00019081** | NA |
| **Maker00006181** | NA |
| **Maker00011567** | biological_process:GO:0009451//RNA modification;cellular_component:GO:0043231//intracellular membrane-bounded organelle;molecular_function:GO:0003723//RNA binding;GO:0004386//helicase activity;GO:0004519//endonuclease activity; |
| **Maker00005255** | biological_process:GO:0008152//metabolic process;molecular_function:GO:0016758//transferase activity, transferring hexosyl groups; |
| **Maker00005528** | NA |
| **Maker00005588** | biological_process:GO:0008152//metabolic process;cellular_component:GO:0043231//intracellular membrane-bounded organelle;molecular_function:GO:0080043//quercetin 3-O-glucosyltransferase activity;GO:0080044//quercetin 7-O-glucosyltransferase activity; |
| **Maker00005729** | biological_process:GO:0008152//metabolic process;cellular_component:GO:0043231//intracellular membrane-bounded organelle;molecular_function:GO:0080043//quercetin 3-O-glucosyltransferase activity;GO:0080044//quercetin 7-O-glucosyltransferase activity; |
| **Maker00006525** | biological_process:GO:0008152//metabolic process;cellular_component:GO:0043231//intracellular membrane-bounded organelle;molecular_function:GO:0080043//quercetin 3-O-glucosyltransferase activity;GO:0080044//quercetin 7-O-glucosyltransferase activity; |
| **Maker00001801** | biological_process:GO:0008152//metabolic process;molecular_function:GO:0016758//transferase activity, transferring hexosyl groups; |
| **Maker00002259** | biological_process:GO:0008152//metabolic process;cellular_component:GO:0043231//intracellular membrane-bounded organelle;molecular_function:GO:0080043//quercetin 3-O-glucosyltransferase activity;GO:0080044//quercetin 7-O-glucosyltransferase activity;GO:0016758//transferase activity, transferring hexosyl groups; |
| **Maker00003306** | NA |
| **Maker00003559** | NA |
| **Maker00003734** | biological_process:GO:0008152//metabolic process;cellular_component:GO:0043231//intracellular membrane-bounded organelle;molecular_function:GO:0080043//quercetin 3-O-glucosyltransferase activity;GO:0080044//quercetin 7-O-glucosyltransferase activity; |
| **Maker00010789** | biological_process:GO:0008152//metabolic process;cellular_component:GO:0043231//intracellular membrane-bounded organelle;molecular_function:GO:0080043//quercetin 3-O-glucosyltransferase activity;GO:0080044//quercetin 7-O-glucosyltransferase activity; |
| **Maker00010826** | biological_process:GO:0008152//metabolic process;cellular_component:GO:0043231//intracellular membrane-bounded organelle;molecular_function:GO:0080043//quercetin 3-O-glucosyltransferase activity;GO:0080044//quercetin 7-O-glucosyltransferase activity; |
| **Maker00007420** | molecular_function:GO:0016740//transferase activity; |
| **Maker00012009** | NA |
| **Maker00015832** | NA |
| **Maker00017966** | NA |
| **Maker00018328** | NA |
| **Maker00006330** | NA |
| **Maker00006346** | biological_process:GO:0019438//aromatic compound biosynthetic process;molecular_function:GO:0008757//S-adenosylmethionine-dependent methyltransferase activity;GO:0008171//O-methyltransferase activity;GO:0046983//protein dimerization activity; |
| **Maker00014127** | biological_process:GO:0019438//aromatic compound biosynthetic process;molecular_function:GO:0008757//S-adenosylmethionine-dependent methyltransferase activity;GO:0008171//O-methyltransferase activity;GO:0046983//protein dimerization activity; |
| **Maker00003028** | biological_process:GO:0008152//metabolic process;cellular_component:GO:0043231//intracellular membrane-bounded organelle;molecular_function:GO:0080043//quercetin 3-O-glucosyltransferase activity;GO:0080044//quercetin 7-O-glucosyltransferase activity; |
| **Maker00003046** | biological_process:GO:0008152//metabolic process;cellular_component:GO:0043231//intracellular membrane-bounded organelle;molecular_function:GO:0080043//quercetin 3-O-glucosyltransferase activity;GO:0080044//quercetin 7-O-glucosyltransferase activity; |
| **Maker00003528** | biological_process:GO:0008152//metabolic process;cellular_component:GO:0043231//intracellular membrane-bounded organelle;molecular_function:GO:0080043//quercetin 3-O-glucosyltransferase activity;GO:0080044//quercetin 7-O-glucosyltransferase activity; |
| **Maker00003765** | biological_process:GO:0008152//metabolic process;cellular_component:GO:0043231//intracellular membrane-bounded organelle;molecular_function:GO:0080043//quercetin 3-O-glucosyltransferase activity;GO:0080044//quercetin 7-O-glucosyltransferase activity;GO:0016758//transferase activity, transferring hexosyl groups; |
| **Maker00011665** | biological_process:GO:0008152//metabolic process;cellular_component:GO:0043231//intracellular membrane-bounded organelle;molecular_function:GO:0080043//quercetin 3-O-glucosyltransferase activity;GO:0080044//quercetin 7-O-glucosyltransferase activity; |
| **Maker00011571** | NA |
| **Maker00014550** | biological_process:GO:0007165//signal transduction;GO:0006952//defense response;cellular_component:GO:0005886//plasma membrane;molecular_function:GO:0043531//ADP binding;GO:0004721//phosphoprotein phosphatase activity; |
| **Maker00003042** | biological_process:GO:0044550//secondary metabolite biosynthetic process;cellular_component:GO:0016020//membrane;GO:0016021//integral component of membrane;molecular_function:GO:0016709//oxidoreductase activity, acting on paired donors, with incorporation or reduction of molecular oxygen, NAD(P)H as one donor, and incorporation of one atom of oxygen;GO:0020037//heme binding;GO:0005506//iron ion binding; |
| **Maker00005942** | cellular_component:GO:0016021//integral component of membrane;molecular_function:GO:0016705//oxidoreductase activity, acting on paired donors, with incorporation or reduction of molecular oxygen;GO:0020037//heme binding;GO:0005506//iron ion binding;GO:0004497//monooxygenase activity; |
| **Maker00011659** | biological_process:GO:0044550//secondary metabolite biosynthetic process;cellular_component:GO:0016020//membrane;GO:0016021//integral component of membrane;molecular_function:GO:0016709//oxidoreductase activity, acting on paired donors, with incorporation or reduction of molecular oxygen, NAD(P)H as one donor, and incorporation of one atom of oxygen;GO:0020037//heme binding;GO:0005506//iron ion binding;GO:0033772//flavonoid 3',5'-hydroxylase activity; |
| **Maker00004902** | molecular_function:GO:0030246//carbohydrate binding;GO:0005524//ATP binding;GO:0004672//protein kinase activity; |
| **Maker00015382** | cellular_component:GO:0016021//integral component of membrane;molecular_function:GO:0030246//carbohydrate binding;GO:0005524//ATP binding;GO:0004672//protein kinase activity; |
| **Maker00013239** | cellular_component:GO:0016021//integral component of membrane;GO:0005886//plasma membrane;molecular_function:GO:0004674//protein serine/threonine kinase activity;GO:0005524//ATP binding; |
| **Maker00013929** | NA |
| **Maker00017661** | cellular_component:GO:0016021//integral component of membrane;molecular_function:GO:0005524//ATP binding;GO:0004672//protein kinase activity; |
| **Maker00018240** | NA |
| **Maker00015596** | NA |
| **Maker00015954** | NA |
| **Maker00017824** | NA |
| **Maker00017912** | NA |
| **Maker00018681** | biological_process:GO:0006468//protein phosphorylation;cellular_component:GO:0016021//integral component of membrane;GO:0005886//plasma membrane;molecular_function:GO:0004674//protein serine/threonine kinase activity;GO:0005524//ATP binding; |
| **Maker00013338** | cellular_component:GO:0016021//integral component of membrane;molecular_function:GO:0016301//kinase activity; |
| **Maker00002802** | biological_process:GO:0016042//lipid catabolic process;molecular_function:GO:0016787//hydrolase activity; |
| **Maker00003015** | biological_process:GO:0016042//lipid catabolic process;molecular_function:GO:0016787//hydrolase activity; |
| **Maker00011619** | biological_process:GO:0016042//lipid catabolic process;molecular_function:GO:0016787//hydrolase activity; |
| **Maker00011909** | biological_process:GO:0016042//lipid catabolic process;molecular_function:GO:0016787//hydrolase activity; |
| **Maker00003608** | molecular_function:GO:0016705//oxidoreductase activity, acting on paired donors, with incorporation or reduction of molecular oxygen;GO:0020037//heme binding;GO:0005506//iron ion binding;GO:0004497//monooxygenase activity; |
| **Maker00014883** | cellular_component:GO:0016021//integral component of membrane;molecular_function:GO:0016705//oxidoreductase activity, acting on paired donors, with incorporation or reduction of molecular oxygen;GO:0020037//heme binding;GO:0050598//taxane 13-alpha-hydroxylase activity;GO:0005506//iron ion binding;GO:0004497//monooxygenase activity; |
| **Maker00016234** | biological_process:GO:0010268//brassinosteroid homeostasis;GO:0016132//brassinosteroid biosynthetic process;GO:0007275//multicellular organism development;GO:0016125//sterol metabolic process;cellular_component:GO:0016021//integral component of membrane;molecular_function:GO:0020037//heme binding;GO:0005506//iron ion binding;GO:0016705//oxidoreductase activity, acting on paired donors, with incorporation or reduction of molecular oxygen;GO:0004497//monooxygenase activity; |
| **Maker00004886** | NA |
| **Maker00009207** | NA |
| **Maker00011037** | NA |
| **Maker00012554** | NA |
| **Maker00013657** | NA |
| **Maker00003133** | NA |
| **Maker00003304** | biological_process:GO:0008152//metabolic process;molecular_function:GO:0003824//catalytic activity; |
| **Maker00007713** | NA |
| **Maker00010459** | NA |
| **Maker00012429** | NA |
| **Maker00005471** | NA |
| **Maker00015116** | NA |
| **Maker00015733** | NA |
| **Maker00016637** | NA |
| **Maker00012062** | NA |
| **Maker00001117** | biological_process:GO:0008643//carbohydrate transport;cellular_component:GO:0016021//integral component of membrane;molecular_function:GO:0015293//symporter activity; |
| **Maker00007972** | biological_process:GO:0035428//hexose transmembrane transport;GO:0046323//glucose import;cellular_component:GO:0016021//integral component of membrane;molecular_function:GO:0005355//glucose transmembrane transporter activity;GO:0005351//sugar:proton symporter activity; |
| **Maker00017364** | biological_process:GO:0008643//carbohydrate transport;cellular_component:GO:0016021//integral component of membrane;molecular_function:GO:0015293//symporter activity; |
| **Maker00001222** | molecular_function:GO:0046872//metal ion binding;GO:0016707//gibberellin 3-beta-dioxygenase activity;GO:0016491//oxidoreductase activity; |
| **Maker00011727** | molecular_function:GO:0102078//methyl jasmonate methylesterase activity;GO:0030795//jasmonate O-methyltransferase activity; |
| **Maker00012718** | molecular_function:GO:0008168//methyltransferase activity; |
| **Maker00011621** | biological_process:GO:0030001//metal ion transport;molecular_function:GO:0046872//metal ion binding; |
| **Maker00011924** | biological_process:GO:0030001//metal ion transport;molecular_function:GO:0046872//metal ion binding; |
| **Maker00006920** | molecular_function:GO:0008270//zinc ion binding;GO:0046029//mannitol dehydrogenase activity;GO:0016491//oxidoreductase activity; |
| **Maker00007586** | molecular_function:GO:0008270//zinc ion binding;GO:0016491//oxidoreductase activity; |
| **Maker00004122** | NA |
| **Maker00017708** | NA |
| **Maker00018174** | cellular_component:GO:0005634//nucleus;GO:0000786//nucleosome;molecular_function:GO:0003677//DNA binding;GO:0046982//protein heterodimerization activity; |
| **Maker00001864** | biological_process:GO:0008152//metabolic process;molecular_function:GO:0016758//transferase activity, transferring hexosyl groups; |
| **Maker00005953** | biological_process:GO:0006032//chitin catabolic process;GO:0016998//cell wall macromolecule catabolic process;GO:0005975//carbohydrate metabolic process;molecular_function:GO:0008061//chitin binding;GO:0004568//chitinase activity; |
| **Maker00007266** | NA |
| **Maker00013855** | NA |
| **Maker00014064** | molecular_function:GO:0016301//kinase activity; |
| **Maker00018508** | biological_process:GO:0044550//secondary metabolite biosynthetic process;cellular_component:GO:0016020//membrane;molecular_function:GO:0016709//oxidoreductase activity, acting on paired donors, with incorporation or reduction of molecular oxygen, NAD(P)H as one donor, and incorporation of one atom of oxygen;GO:0020037//heme binding;GO:0005506//iron ion binding; |
| **Maker00018543** | biological_process:GO:0044550//secondary metabolite biosynthetic process;cellular_component:GO:0016020//membrane;molecular_function:GO:0016709//oxidoreductase activity, acting on paired donors, with incorporation or reduction of molecular oxygen, NAD(P)H as one donor, and incorporation of one atom of oxygen;GO:0020037//heme binding;GO:0005506//iron ion binding; |
| **Maker00007614** | NA |
| **Maker00009305** | NA |
| **Maker00003802** | biological_process:GO:0055074//calcium ion homeostasis;molecular_function:GO:0005432//calcium:sodium antiporter activity;GO:0005509//calcium ion binding; |
| **Maker00006913** | cellular_component:GO:0016021//integral component of membrane; |
| **Maker00009200** | NA |
| **Maker00009243** | NA |
| **Maker00005985** | cellular_component:GO:0016021//integral component of membrane; |
| **Maker00006364** | NA |
| **Maker00018288** | cellular_component:GO:0016021//integral component of membrane;molecular_function:GO:0005215//transporter activity; |
| **Maker00018825** | cellular_component:GO:0016021//integral component of membrane;molecular_function:GO:0005215//transporter activity; |
| **Maker00010120** | cellular_component:GO:0016021//integral component of membrane; |
| **Maker00016944** | cellular_component:GO:0016021//integral component of membrane; |
| **Maker00001195** | NA |
| **Maker00014548** | NA |
| **Maker00004085** | molecular_function:GO:0004190//aspartic-type endopeptidase activity; |
| **Maker00008107** | molecular_function:GO:0032440//2-alkenal reductase [NAD(P)] activity;GO:0005509//calcium ion binding; |
| **Maker00001845** | cellular_component:GO:0016021//integral component of membrane;GO:0005802//trans-Golgi network;GO:0005886//plasma membrane; |
| **Maker00006694** | cellular_component:GO:0016021//integral component of membrane;GO:0005802//trans-Golgi network;GO:0005886//plasma membrane; |
| **Maker00017237** | NA |
| **Maker00000567** | biological_process:GO:0030042//actin filament depolymerization;cellular_component:GO:0015629//actin cytoskeleton;molecular_function:GO:0003779//actin binding; |
| **Maker00005306** | NA |
| **Maker00000188** | NA |
| **Maker00008460** | NA |
| **Maker00000044** | cellular_component:GO:0005634//nucleus;molecular_function:GO:0003676//nucleic acid binding; |
| **Maker00010279** | biological_process:GO:0010200//response to chitin;cellular_component:GO:0005634//nucleus;molecular_function:GO:0003676//nucleic acid binding; |
| **Maker00004559** | cellular_component:GO:0005737//cytoplasm;molecular_function:GO:0004791//thioredoxin-disulfide reductase activity; |
| **Maker00002041** | biological_process:GO:0006486//protein glycosylation;cellular_component:GO:0016021//integral component of membrane;molecular_function:GO:0016757//transferase activity, transferring glycosyl groups; |
| **Maker00014015** | NA |
| **Maker00004067** | NA |
| **Maker00009240** | biological_process:GO:0006351//transcription, DNA-templated;cellular_component:GO:0005634//nucleus;molecular_function:GO:0003677//DNA binding;GO:0003700//DNA binding transcription factor activity; |
| **Maker00008572** | molecular_function:GO:0005509//calcium ion binding; |
| **Maker00000439** | NA |
| **Maker00018889** | NA |
| **Maker00009216** | NA |
| **Maker00001708** | NA |
| **Maker00011100** | NA |
| **Maker00011453** | cellular_component:GO:0016021//integral component of membrane;molecular_function:GO:0022857//transmembrane transporter activity; |
| **Maker00016256** | NA |
| **Maker00007920** | biological_process:GO:0008033//tRNA processing; |
| **Maker00001694** | cellular_component:GO:0016021//integral component of membrane; |
| **Maker00008047** | cellular_component:GO:0016021//integral component of membrane; |
| **Maker00009557** | biological_process:GO:0071712//ER-associated misfolded protein catabolic process;GO:0042787//protein ubiquitination involved in ubiquitin-dependent protein catabolic process;cellular_component:GO:0016021//integral component of membrane;GO:0036513//Derlin-1 retrotranslocation complex;molecular_function:GO:0046872//metal ion binding;GO:0044390//ubiquitin-like protein conjugating enzyme binding;GO:1904264//ubiquitin protein ligase activity involved in ERAD pathway; |
| **Maker00013106** | biological_process:GO:0071712//ER-associated misfolded protein catabolic process;GO:0042787//protein ubiquitination involved in ubiquitin-dependent protein catabolic process;cellular_component:GO:0016021//integral component of membrane;GO:0036513//Derlin-1 retrotranslocation complex;molecular_function:GO:0046872//metal ion binding;GO:0044390//ubiquitin-like protein conjugating enzyme binding;GO:1904264//ubiquitin protein ligase activity involved in ERAD pathway; |
| **Maker00005326** | NA |
| **Maker00016164** | molecular_function:GO:0003723//RNA binding; |
| **Maker00008995** | biological_process:GO:0006351//transcription, DNA-templated;cellular_component:GO:0005634//nucleus;molecular_function:GO:0003677//DNA binding;GO:0003700//DNA binding transcription factor activity; |
| **Maker00015302** | NA |
| **Maker00002818** | NA |
| **Maker00018658** | NA |
| **Maker00010163** | biological_process:GO:0008152//metabolic process;cellular_component:GO:0043231//intracellular membrane-bounded organelle;molecular_function:GO:0080043//quercetin 3-O-glucosyltransferase activity;GO:0080044//quercetin 7-O-glucosyltransferase activity; |
| **Maker00007308** | molecular_function:GO:0003677//DNA binding;GO:0003700//DNA binding transcription factor activity; |
| **Maker00016862** | biological_process:GO:0008283//cell proliferation;cellular_component:GO:0005576//extracellular region;molecular_function:GO:0008083//growth factor activity; |
| **Maker00005487** | NA |
| **Maker00016826** | molecular_function:GO:0016788//hydrolase activity, acting on ester bonds; |
| **Maker00014047** | NA |
| **Maker00007187** | biological_process:GO:0045454//cell redox homeostasis;cellular_component:GO:0005623//cell;molecular_function:GO:0015035//protein disulfide oxidoreductase activity;GO:0009055//electron transfer activity; |
| **Maker00007793** | cellular_component:GO:0016021//integral component of membrane; |
| **Maker00009232** | biological_process:GO:0000160//phosphorelay signal transduction system;cellular_component:GO:0005622//intracellular;molecular_function:GO:0016301//kinase activity; |
| **Maker00003973** | NA |
| **Maker00002921** | NA |
| **Maker00002561** | NA |
| **Maker00007754** | biological_process:GO:0006357//regulation of transcription from RNA polymerase II promoter;GO:0030154//cell differentiation;cellular_component:GO:0005634//nucleus;molecular_function:GO:0000981//RNA polymerase II transcription factor activity, sequence-specific DNA binding;GO:0043565//sequence-specific DNA binding;GO:0044212//transcription regulatory region DNA binding;GO:0001135//transcription factor activity, RNA polymerase II transcription factor recruiting; |

## Supplementary Table 13. The results of whole genome collinearity analysis.

| **Species vs Species** | **Gene pair number** |
| --- | --- |
| *K. obovata vs R. apiculata* | 11,010 |
| *K. obovata vs K. obovata* | 3,840 |
| *R. apiculata vs R. apiculata* | 4,646 |
| *K. obovata vs V. vinifera* | 10,893 |

## Supplementary Table 14. List of MADS-box genes identified in *K. obovata* and *R. apiculata*.

| Gene ID | Name | Type | Subfamily | Pseudogene | Protein length (aa) | ORF (bp) |
| --- | --- | --- | --- | --- | --- | --- |
| Gene.178810 | KoBs | MIKCc | Bs |  |  |  |
| Maker00017934 | KoAG | MIKCc | C/D |  | 338 | 271 |
| Maker00008916 | KoTM.1 | MIKCc | SOC1 |  | 328 | 284 |
| Maker00014303 | KoTM.2 | MIKCc | SOC1 |  | 234 | 208 |
| Maker00018317 | KoTM.3 | MIKCc | SOC1 |  | 256 | 215 |
| Maker00009412 | KoTM.4 | MIKCc | SOC1 |  | 260 | 219 |
| Maker00013628 | KoTM.5 | MIKCc | SOC1 |  | 252 | 219 |
| Maker00002724 | KoAP.1 | MIKCc | A |  | 271 | 243 |
| Maker00000561 | KoAP.2 | MIKCc | A |  | 292 | 248 |
| Maker00009974 | KoAP.3 | MIKCc | A |  | 306 | 251 |
| Maker00001865 | KoAP.4 | MIKCc | A |  | 285 | 254 |
| Maker00008198 | KoAP.5 | MIKCc | A |  | 162 | 142 |
| Gene.243203 | KoAGL6 | MIKCc | AGL6 |  | 244 | 210 |
| contig24 1508784 | KoAGL15 | MIKCc | AGL15 |  | 224 | 252 |
| Maker00002385 | KoAGL9.1 | MIKCc | E |  | 162 | 242 |
| Maker00010213 | KoAGL9.2 | MIKCc | E |  | 290 | 243 |
| Maker00007712 | KoAGL9.3 | MIKCc | E |  | 286 | 247 |
| Maker00001812 | KoAGL9.4 | MIKCc | E |  | 306 | 259 |
| Maker00006717 | KoAGL9.5 | MIKCc | E |  | 200 | 172 |
| Maker00002483 | KoAGL9.6 | MIKCc | E |  | 283 | 246 |
| Maker00012501 | KoAP3.1 | MIKCc | B-AP3 |  | 270 | 204 |
| Maker00007295 | KoAP3.2 | MIKCc | B-AP3 |  | 280 | 213 |
| Maker00011580 | KoPI | MIKCc | B_PI |  | 204 | 168 |
| Maker00005329 | KoANR1.1 | MIKCc | ANR1 |  | 307 | 255 |
| Maker00000686 | KoANR1.2 | MIKCc | ANR1 |  | 238 | 192 |
| Maker00012093 | KoAGL12 | MIKCc | AGL12 |  | 260 | 208 |
| Maker00005621 | KoSVP.1 | MIKCc | SVP |  | 669 | 619 |
| Maker00002818 | KoSVP.2 | MIKCc | SVP |  | 275 | 229 |
| Maker00010868 | KoMP.1 | MIKC* |  |  | 345 | 352 |
| Maker00008975 | KoMP.2 | MIKC* |  |  | 571 | 469 |
| Maker00000181 | KoMP.3 | MIKC* |  |  | 448 | 374 |
| Maker00006588 | KoMP.4 | MIKC* |  |  | 255 | 217 |
| Gene.227210 | KoMA.1 | Type I | Mα |  | 136 | 109 |
| Gene.61034 | KoMA.2 | Type I | Mα |  | 131 | 109 |
| Maker00005698 | KoMA.3 | Type I | Mα |  | 310 | 234 |
| Gene.218253 | KoMA.4 | Type I | Mα |  | 291 | 232 |
| Gene.227212 | KoMA.5 | Type I | Mα |  | 167 | 157 |
| Gene.227213 | KoMA.6 | Type I | Mα |  | 365 | 356 |
| Gene.204823 | KoMB.1 | Type I | Mb |  | 223 | 245 |
| Contig25 5554650-5553790 | KoMC.1 | Type I | Mγ |  | 287 | 246 |
| Contig17 71284-72075 | KoMC.2 | Type I | Mγ |  | 264 | 230 |
| Contig4 5074019-5074834 | KoMC.3 | Type I | Mγ |  | 272 | 240 |
| contig17 6697954-6698649 | KoMC.4 | Type I | Mγ |  | 230 | 203 |
| contig32 1489006-1488008 | KoMC.5 | Type I | Mγ |  | 332 | 294 |
| Gene.165291 | KoMA.7 | Type I |  | V |  |  |

| Gene ID | Name | Type | Subfamily | Pseudogene | Protein length (aa) | ORF (bp) |
| --- | --- | --- | --- | --- | --- | --- |
| RA_21148 | RaAG.1 | MIKCc | C/D |  | 312 | 250 |
| RA_23069 | RaAG.2 | MIKCc | C/D |  | 285 | 228 |
| RA_23050 | RaAG.3 | MIKCc | C/D |  | 302 | 242 |
| RA_00873 | RaTM.1 | MIKCc | SOC1 |  | 257 | 222 |
| RA_07969 | RaTM.2 | MIKCc | SOC1 |  | 787 | 621 |
| RA_13010 | RaTM.3 | MIKCc | SOC1 |  | 246 | 214 |
| RA_24864 | RaTM.4 | MIKCc | SOC1 |  | 263 | 223 |
| RA_14202 | RaTM.5 | MIKCc | SOC1 |  | 253 | 215 |
| RA_14203 | RaTM.6 | MIKCc | SOC1 |  | 237 | 207 |
| RA_06420 | RaTM.7 | MIKCc | SOC1 |  | 246 | 218 |
| RA_14932 | RaAP.1 | MIKCc | A |  | 280 | 244 |
| RA_15058 | RaAP.2 | MIKCc | A |  | 285 | 242 |
| RA_12726 | RaAP.3 | MIKCc | A |  | 235 | 211 |
| RA_21304 | RaAP.4 | MIKCc | A |  | 284 | 262 |
| RA_07503 | RaAP.5 | MIKCc | A |  | 280 | 251 |
| RA_00876 | RaAGL6 | MIKCc | AGL6 |  | 291 | 243 |
| RA_14931 | RaAGL9.1 | MIKCc | E |  | 290 | 243 |
| RA_12725 | RaAGL9.2 | MIKCc | E |  | 288 | 244 |
| RA_21305 | RaAGL9.3 | MIKCc | E |  | 290 | 246 |
| RA_14626 | RaAGL9.4 | MIKCc | E |  | 275 | 244 |
| RA_16963 | RaAGL15.1 | MIKCc | AGL15 |  | 302 | 255 |
| RA_24346 | RaAGL15.2 | MIKCc | AGL15 |  | 303 | 250 |
| RA_11944 | RaBs | MIKCc | Bs |  | 345 | 296 |
| RA_10871 | RaAP3.1 | MIKCc | B-AP3 |  | 294 | 226 |
| RA_02492 | RaAP3.2 | MIKCc | B-AP3 |  | 277 | 227 |
| RA_17015 | RaPI | MIKCc | B_PI |  | 270 | 223 |
| RA_19141 | RaANR1.1 | MIKCc | ANR1 |  | 318 | 259 |
| RA_24070 | RaANR1.2 | MIKCc | ANR1 |  | 238 | 193 |
| RA_21146 | RaAGL12 | MIKCc | AGL12 |  | 214 | 180 |
| RA_03270 | RaSVP.1 | MIKCc | SVP |  | 234 | 216 |
| RA_21689 | RaSVP.2 | MIKCc | SVP |  | 258 | 225 |
| RA_02417 | RaSVP.3 | MIKCc | SVP |  | 264 | 229 |
| RA_23303 | RaMP.1 | MIKC* |  |  | 433 | 353 |
| RA_17752 | RaMP.2 | MIKC* |  |  | 431 | 358 |
| RA_14861 | RaMP.3 | MIKC* |  |  | 433 | 365 |
| RA_03174 | RaMB.1 | Type I | Mγ |  | 426 | 333 |
| RA_11252 | RaMB.2 | Type I | Mγ |  | 657 | 491 |
| RA_17478 | RaMB.3 | Type I | Mb |  | 324 | 256 |
| RA_10562 | RaMB.5 | Type I | Mγ |  | 446 | 352 |
| RA_20650 | RaMB.4 | Type I | Mγ |  | 429 | 311 |
| RA_18973 | RaMB.6 | Type I | Mb |  | 510 | 379 |
| RA_24483 | RaMC.1 | Type I | Mγ |  | 304 | 225 |
| RA_09212 | RaMC.2 | Type I | Mγ |  | 589 | 451 |
| RA_09240 | RaMC.3 | Type I | Mγ |  | 440 | 334 |
| RA_20431 | RaMC.4 | Type I | Mγ |  | 337 | 261 |
| RA_22062 | RaMC.5 | Type I | Mγ |  | 293 | 214 |
| RA_16765 | RaMC.6 | Type I | Mγ |  | 374 | 278 |
| RA_23203 | RaMA.1 | Type I | Mα |  | 250 | 183 |
| RA_19106 | RaMA.2 | Type I | Mα |  | 254 | 223 |
| RA_19107 | RaMA.3 | Type I | Mα |  | 266 | 230 |
| RA_03282 | RaMA.4 | Type I | Mα |  | 250 | 213 |
| RA_06824 | RaMA.5 | Type I | Mα |  | 286 | 226 |
| RA_06825 | RaMA.6 | Type I | Mα |  | 222 | 195 |
| RA_19171 | RaMA.7 | Type I | Mα |  | 270 | 218 |
| RA_19172 | RaMA.8 | Type I | Mα |  | 282 | 225 |
| RA_04109 | RaMA.9 | Type I | Mα |  | 290 | 221 |
| RA_14294 | RaMA.10 | Type I | Mα |  | 180 | 149 |
| RA_06940 | RaMA.11 | Type I | Mα |  | 332 | 250 |
| RA_09726 | RaMA.12 | Type I | Mα |  | 314 | 260 |
| RA_10851 | RaMA.13 | Type I | Mα |  | 234 | 213 |
| RA_11847 | RaMA.14 | Type I | Mα |  | 193 | 181 |
| RA_11851 | RaMA.15 | Type I | Mα |  | 225 | 190 |
| RA_17665 | RaMA.16 | Type I | Mα |  | 233 | 181 |
| RA_21409 | RaMA.17 | Type I | Mα |  | 626 | 443 |
| RA_13643 | RaMA.18 | Type I | Mα |  | 476 | 360 |
| RA_18571 | RaMA.19 | Type I | Mα |  | 419 | 320 |
| RA_10609 | RaMP.4 | MIKC* |  | V |  |  |
| RA_19118 | RaSVP.4 | MIKCc | SVP | V |  |  |
| RA_13632 | PeMA1 | Type I | Mα | V |  |  |

The gene ID that starts with ‘Maker’ and ‘contig’is the gene that was searched in the *K. obovata* genome.

The Gene ID beginning with ‘Gene’ is the gene found in the *K. obovata* transcriptome.

The gene ID starting with ‘RA’ is a gene searched in the *R. apiculata* genome.

## Supplementary Table 15. List of reference R genes from different species.

| **Plant** | **Gene** | **Type** |
| --- | --- | --- |
| Arabidopsis thaliana | RPS5 | CC-NBS-LRR |
|  | RPS2 |  |
|  | HRT |  |
|  | RPP13 |  |
|  | RPM1 |  |
|  | AT1G12220 |  |
|  | AT4G26090 |  |
|  | AT5G43470 |  |
|  | AT3G46530 |  |
|  | AT3G07040 |  |
|  | ADR1 | RPW8-NL |
|  | Arabidopsis thaliana |  |
|  | RPP1 | TNL |
|  | RPP5 |  |
|  | TNTNL |  |
|  | RPS1 |  |
|  | AT4G36140 |  |
|  | \| AT4G16950 \| \| --- \| |  |
|  | AT5G45050 |  |
|  | AT1G66090 |  |
|  | RAC1 |  |
|  | RPP4 |  |
|  | AT1G27170 |  |
|  | AT5G36930 |  |
|  | AT1G27180 |  |
|  | AT5G45050 |  |
|  | AT1G50180 | CN |
|  | AT5G66910 | CNL |
|  | AT4G33300 |  |
|  | AT4G09360 | NL |
| Oryza sativa Indica Group | Pi2 | CNL |
|  | Piz-t |  |
|  | Pikm1-TS |  |
|  | Pikp-2 |  |
|  | PIB |  |

This file contains the list of the functional R genes and proteins used as references in the phylogenetic trees.
